# Mediator Acts Upstream of the Transcriptional Activator Gal4

**DOI:** 10.1371/journal.pbio.1001290

**Published:** 2012-03-27

**Authors:** Keven Ang, Gary Ee, Edwin Ang, Elvin Koh, Wee Leng Siew, Yu Mun Chan, Sabrina Nur, Yee Sun Tan, Norbert Lehming

**Affiliations:** Department of Microbiology, Yong Loo Lin School of Medicine, National University of Singapore, Singapore; UMass Medical School, United States of America

## Abstract

The proteasome inhibitor MG132 had been shown to prevent galactose induction of the *S. cerevisiae GAL1* gene, demonstrating that ubiquitin proteasome-dependent degradation of transcription factors plays an important role in the regulation of gene expression. The deletion of the gene encoding the F-box protein Mdm30 had been reported to stabilize the transcriptional activator Gal4 under inducing conditions and to lead to defects in galactose utilization, suggesting that recycling of Gal4 is required for its function. Subsequently, however, it was argued that Gal4 remains stably bound to the enhancer under inducing conditions, suggesting that proteolytic turnover of Gal4 might not be required for its function. We have performed an alanine-scanning mutagenesis of ubiquitin and isolated a galactose utilization-defective ubiquitin mutant. We have used it for an unbiased suppressor screen and identified the inhibitor Gal80 as a suppressor of the transcriptional defects of the ubiquitin mutant, indicating that the protein degradation of the inhibitor Gal80, and not of the activator Gal4, is required for galactose induction of the *GAL* genes. We also show that in the absence of Gal80, Mdm30 is not required for Gal4 function, strongly supporting this hypothesis. Furthermore, we have found that Mediator controls the galactose-induced protein degradation of Gal80, which places Mediator genetically upstream of the activator Gal4. Mediator had originally been isolated by its ability to respond to transcriptional activators, and here we have discovered a leading role for Mediator in the process of transcription. The protein kinase Snf1 senses the inducing conditions and transduces the signal to Mediator, which initiates the degradation of the inhibitor Gal80 with the help of the E3 ubiquitin ligase SCF^Mdm30^. The ability of Mediator to control the protein degradation of transcriptional inhibitors indicates that Mediator is actually able to direct its own recruitment to gene promoters.

## Abstract 2

We show that Mediator, a protein originally isolated on the basis of its ability to respond to transcriptional activators, and thought to be regulated by an activator, can also be the master that controls the activator.

## Author Summary

The expression levels of proteins are tightly regulated, not only via their production but also via their degradation. Genes are transcribed only if their encoded proteins are required by the environmental or developmental conditions of a cell, and once a certain protein is no longer needed, it is rapidly degraded by the ubiquitin proteasome system (UPS). Transcriptional activators appeared to contradict this simple economic principle, as it had been claimed that they had to be degraded in order to function. The claim was based upon a correlation: if the degradation of an activator was prevented by drugs or mutations in the UPS, the activator became stable but also nonfunctional. We have now shown that it is not the activator itself but its inhibitor that is the functionally relevant target of the UPS. Furthermore, we have found that the degradation of the inhibitor is controlled by a protein complex called Mediator. The activator is known to recruit Mediator to gene promoters, where Mediator assists RNA polymerase in initiating transcription. Mediator was always considered to be completely under the control of the activator; however, we observe that by regulating the degradation of the inhibitor, Mediator is also able to control the activator and thereby to orchestrate its own recruitment to gene promoters.

## Introduction

Cells regulate the expression of their genes according to requirement [1]. Activators recruit chromatin-remodeling or chromatin-modifying complexes that change the structure of chromatin to promote transcription [2],[3], while repressors recruit chromatin-modifying complexes that change the structure of chromatin to prevent transcription [4],[5]. Repressors also bind directly to activators and prevent the recruitment of the transcription machinery [6]. According to the reverse recruitment hypothesis [7], the transcription factors do not move to the highly transcribed genes, but the highly transcribed genes move to the gene expression machines (GEMs), which are protein complexes with fixed locations in the nuclear periphery. GEMs, which host all transcription factors that are required for gene expression from RNA Polymerase to RNA capping, splicing, poly-adenylation, and export factors [8], are associated with the nuclear pores, and the mature mRNAs, once produced at the GEM, are immediately exported out of the nucleus to be translated at the ribosomes of the rough endoplasmic reticulum [7].

The *Saccharomyces cerevisiae GAL* genes are a paradigm for transcriptional regulation in eukaryotes [9]. In cells grown with glucose, Gal80 binds to Gal4 and blocks its activation function [10], while Mig1 binds to an upstream silencer and recruits the general repressor Tup1 to prevent gene expression [11]. Upon the switch to galactose media, Snf1 phosphorylates Mig1, causing its translocation from the nucleus to the cytoplasm [12], while Gal80 dissociates from Gal4 [13] and is sequestered in the cytoplasm by Gal3 [14], leaving Gal4 free to activate the *GAL* genes, which are required for galactose utilization [7].

Proteolytic stability of transcription factors offers an intriguing possibility for the eukaryotic cell to control gene expression [15]. Ubiquitin proteasome-dependent degradation (UPD) of activators and repressors plays an important role in gene regulation [16], and treatment of *S. cerevisiae* cells with the proteasome inhibitor MG132 abolished galactose induction of the *GAL1* gene [17]. Ubiquitin is a small protein of 76 amino acids that is transferred by E3 ubiquitin ligases to proteins to be targeted for degradation by the 26S proteasome [18]. F-box proteins confer substrate specificity to SCF (Skip1-Cullin-F-box protein) E3 ubiquitin ligases [19]. When cells are grown with galactose, an SCF E3 ubiquitin ligase containing the F-box protein Mdm30, SCF^Mdm30^, ubiquitinates Gal4 [20]. The deletion of *MDM30* stabilizes Gal4 under inducing conditions and leads to defects in galactose utilization, suggesting that recycling of Gal4 is required for its transcriptional activator function [20]. Subsequently, however, it was argued that Gal4 remains stably bound to the enhancer under inducing conditions, suggesting that proteolytic turnover of Gal4 might not be required for its function [21]–[23]. Previously, it had been shown that mono-ubiquitination protected Gal4 from the promoter-stripping activity of proteasomal ATPases [24]–[26], suggesting a role for ubiquitin in transcriptional activation other than protein degradation. Recently, it has been reported that the proteolytic stability of Mediator subunits is inversely correlated with their ability to activate transcription when fused to a DNA-binding domain [27].

Mediator is a complex of more than 20 proteins that is conserved from yeast to man [28]. It was discovered by its ability to respond to transcriptional activators in vivo and in vitro [29]. Genome-wide gene expression studies with temperature-sensitive alleles have shown that Mediator is required for the transcription of nearly all RNA Polymerase II–dependent genes in yeast [30]. Mediator interacts directly with activators, General Transcription Factors, and RNA Polymerase II [31]. In higher eukaryotes, Mediator facilitates a DNA loop between enhancer and basal promoter via its interaction with cohesin [32]. In addition, Mediator affects steps that are downstream of the recruitment of RNA Polymerase II to the core promoter, as Med26-containing metazoan Mediator switches RNA Polymerase into the productive transcription elongation mode by an interaction of Med26 with TBP (TATA-binding protein) and the CTD (C-terminal domain of RNA Polymerase II) kinase P-TEFb [33]. Mediator also modifies chromatin via its own CDK8 subunit, which phosphorylates histone H3S10, and by its interaction with histone acetyl- and methyltransferases [34],[35]. Metazoan Mediator plays important roles in neurogenesis, cancer formation, and stem cell proliferation [31]. All of these reported functions of Mediator are genetically downstream of transcriptional activators. Here, we have found that Mediator additionally is able to act upstream of the transcriptional activator Gal4 by controlling the ubiquitin-mediated protein degradation of the inhibitor Gal80. In the absence of Gal80, Gal4 is free to recruit Mediator to the promoter of the *GAL* genes. Therefore, Mediator actually orchestrates its own recruitment to the *GAL* promoters upon galactose induction.

## Results

### Gal80 Is Stable in a *gal^−^* Ubiquitin Mutant Strain

**Figure 1:**
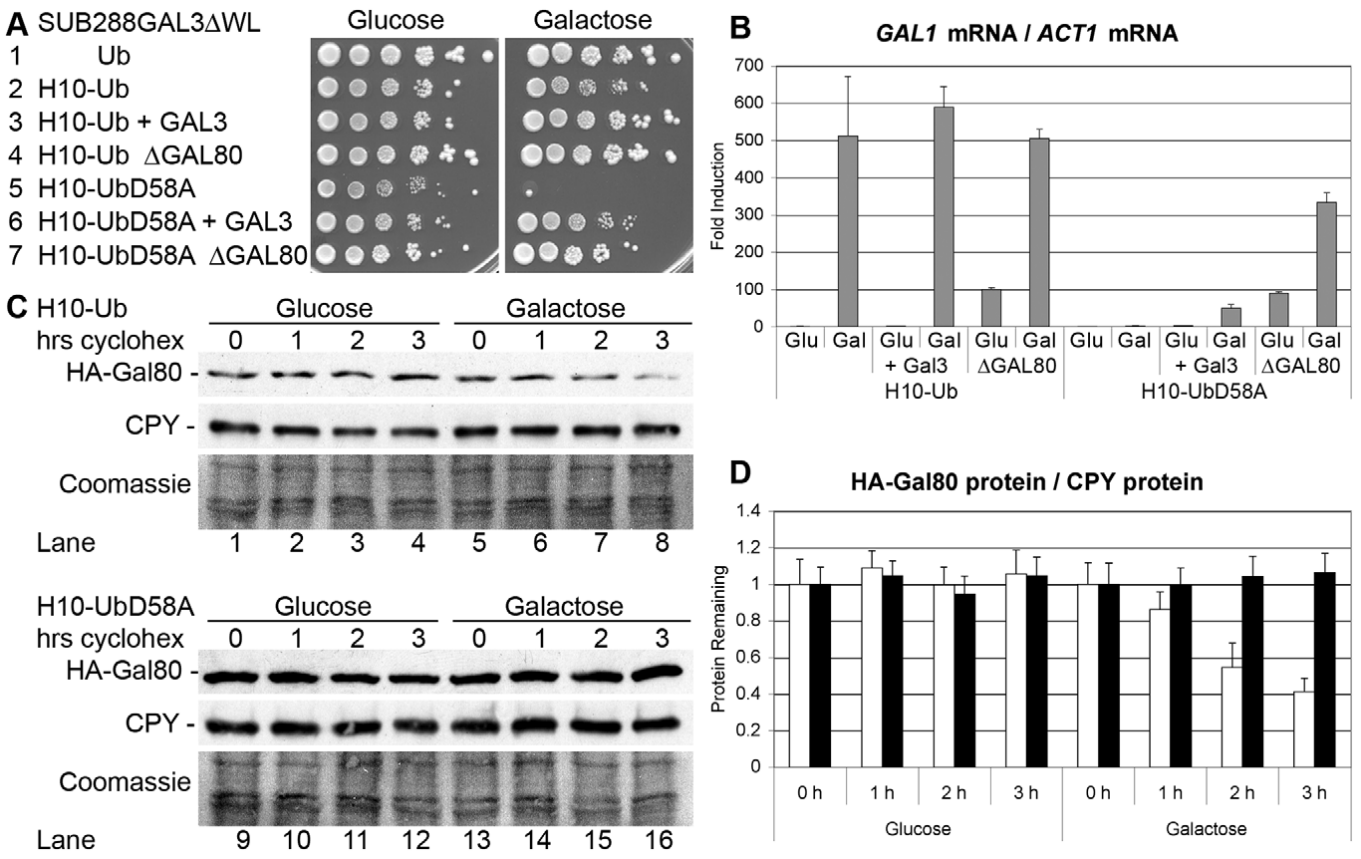
Gal80 is stable in galactose-induced *H_10_UbD58A* cells. (A) *SUB288GAL3ΔWL* cells expressing the indicated ubiquitin derivative in place of endogenous ubiquitin were 10-fold serially diluted, titrated onto glucose and onto galactose plates, and incubated at 28°C for 6 d. Cells in lines 3 and 6 over-expressed Gal3 from the *ACT1* promoter, while cells in lines 4 and 7 lacked *GAL80*. (B) *SUB288GAL3ΔL* cells expressing H_10_Ub and H_10_UbD58A in place of endogenous ubiquitin as indicated were grown in glucose liquid media to OD_600 nm_ = 1 (Glu) and induced with galactose liquid media for 6 h (Gal). Cells over-expressed Gal3 from the *ACT1* promoter or lacked *GAL80* as indicated. Total RNA was isolated and the amount of *GAL1* mRNA relative to *ACT1* mRNA was determined with reverse transcription-coupled real-time PCR. The value determined for cells expressing H_10_Ub grown with glucose was set as 1, and the error bars indicate the standard deviations between three replicates. (C) *SUB288GAL3ΔL* cells expressing H_10_Ub (lanes 1 to 8) or H_10_UbD58A (lanes 9 to 16) in place of endogenous ubiquitin were transformed with the single-copy vector *RS316* expressing HA-Gal80 under the control of the *ACT1* promoter. Cells were grown in glucose liquid media to OD_600 nm_ = 1 and induced with galactose liquid media for 1 h. Cycloheximide was added at time = 0 and the amount of Gal80 protein remaining in the cells after the indicated number of hours was determined by Western blot with the help of an anti-hemagglutinin (HA) antibody (upper panels). The membranes were stripped and reprobed with an anti-carboxypeptidase Y (CPY) antibody (middle panels), followed by a second stripping and staining with Coomassie Blue as loading controls (lower panels). (D) The ratio of the amount of HA-Gal80 protein to CPY protein in *H_10_-Ub* cells (white bars) and *H_10_-UbD58A* cells (black bars) for each time point in part C was determined with Image J. The ratio of the band intensities before the addition of cycloheximide (time = 0) was set as 1, and the error bars indicate the deviations between duplicates.

The role of ubiquitin proteasome-dependent protein degradation in the transcriptional regulation of the *GAL* genes has been controversial [19]–[22]. We performed an alanine-scanning mutagenesis of ubiquitin in order to isolate galactose-utilization defective (*gal* ^−^) mutant strains and use these for unbiased multi-copy suppressor screens. However, no ubiquitin single point mutant displaying the *gal* ^−^ phenotype was isolated (Figure S1, even lanes; Figure S2). The addition of an N-terminal tag can sometimes enhance the phenotype of point mutants, and so we fused a stretch of 10 N-terminal histidines to all ubiquitin mutant proteins. *S. cerevisiae* cells expressing H_10_UbF4A, H_10_UbK6A, H_10_UbI13A, H_10_UbR42A, H_10_UbF45A, H_10_UbD58A, and H_10_UbT66A in the place of endogenous ubiquitin displayed growth defects on galactose plates containing the respiration inhibitor Antimycin A (AA; Figure S1, lanes 5, 11, 23, 85, 87, 105, 119; Figure S2). The presence of the respiration inhibitor AA requires the cells to metabolize more galactose molecules in order to form colonies, which serves to translate defects in the transcriptional activation of the *GAL* genes into stronger growth defects on galactose plates. The *H_10_UbD58A* mutant strain was also unable to grow on galactose plates in the absence of AA (Figure 1A, line 5), and it was transformed with a multi-copy library of *S. cerevisiae* genomic DNA fragments [36]. Gal3 was isolated by its ability to confer growth to the *H_10_UbD58A* mutant strain on galactose plates upon over-expression (Figure 1A, line 6). The over-expression of Gal3 also dosage-compensated the *gal* ^−^ phenotype of the other *H_10_Ub* mutant strains (Figure S3, compare odd and even lanes; the *H_10_UbF4A* mutant strain was barely viable and was excluded from further studies). Gal3 sequesters Gal80 in the cytoplasm upon galactose induction [10], and our finding that the over-expression of Gal3 suppressed the *gal* ^−^ phenotype of the *H_10_Ub* mutant strains indicated that ubiquitin-mediated protein degradation of Gal80 could be required for galactose induction of the *GAL* genes and that the *gal* ^−^ phenotype of these *H_10_Ub* mutant strains might have been caused by excess Gal80. Consistently, the additional gene deletion of *GAL80* suppressed the *gal* ^−^ phenotype of the *H_10_UbD58A* mutant (Figure 1A, line 7) and of the other *H_10_Ub* mutant strains (Figure S4). Reverse transcription coupled with real-time PCR quantification revealed that galactose induction of *GAL1* mRNA relative to *ACT1* mRNA was abolished in the *H_10_UbD58A* strain and that the over-expression of Gal3 and the additional gene deletion of *GAL80* (partially) restored galactose induction (Figure 1B). We performed chase assays with the protein biosynthesis inhibitor cycloheximide and found that HA-Gal80 was indeed degraded in galactose-induced *H_10_Ub* cells (Figure 1C, lanes 5 to 8; Figure 1D, white bars). Importantly, HA-Gal80 had become stable in galactose-induced *H_10_UbD58A* mutant cells (Figure 1C, lanes 13 to 16; Figure 1D, black bars) as well as in the other *gal* ^−^ *H_10_Ub* mutants strains (Figure S5), suggesting that the galactose-stimulated protein degradation of Gal80 is necessary for transcriptional activation of the *GAL* genes. Our finding that the additional gene deletion of *GAL80* suppressed the *gal* ^−^ phenotype of the *H_10_Ub* mutant strains provides genetic evidence that the failure to degrade Gal80 had been the cause (and not the consequence) of the *gal* ^−^ phenotype of the *H_10_Ub* mutant strains.

### Galactose Induction Requires Gal80 Degradation

**Figure 2:**
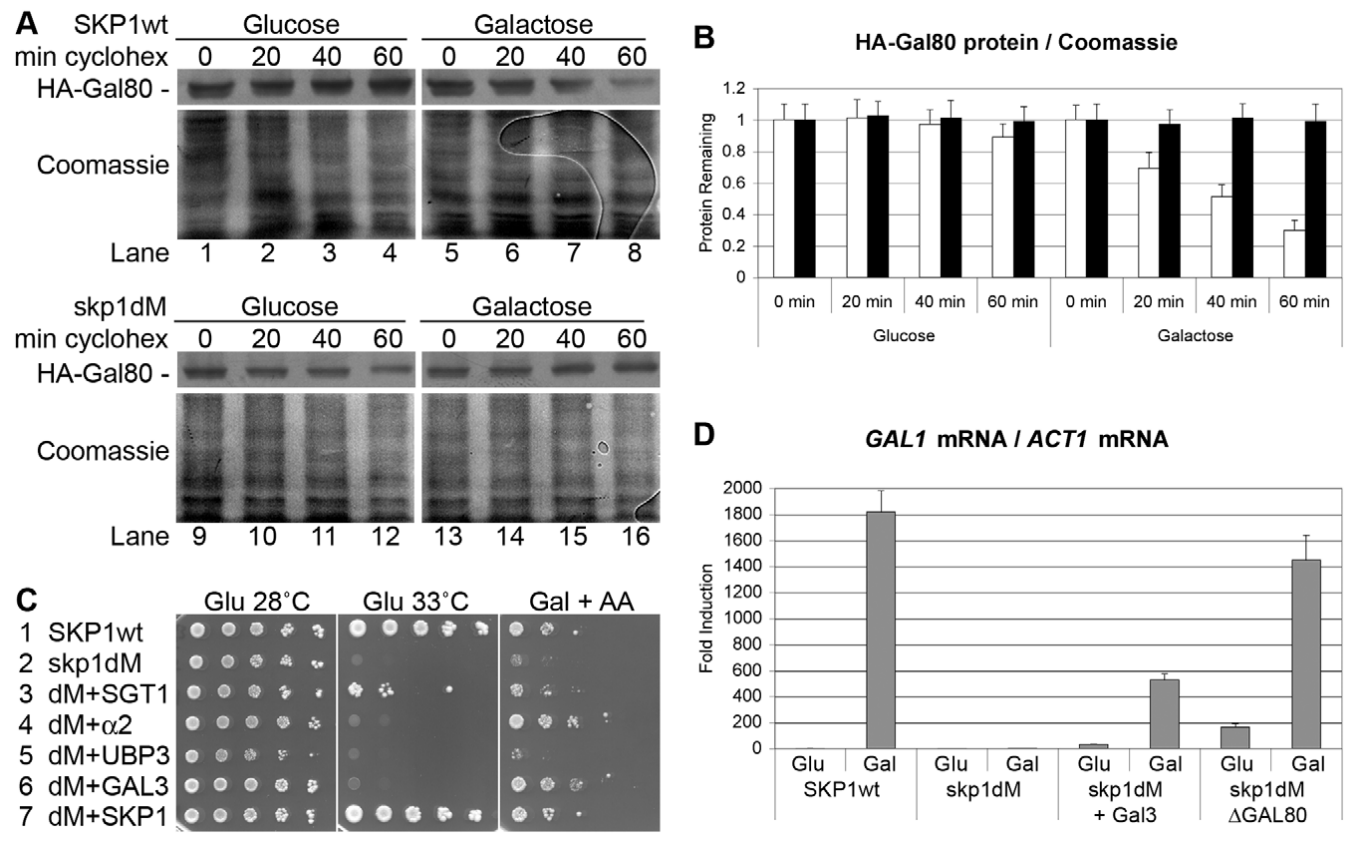
Galactose induction requires SCF-mediated Gal80 degradation. (A) *JD52* cells whose chromosomal *SKP1* gene had been replaced by *HIS3* and that expressed Nub-Skp1 (SKP1wt) or Nub-Skp1V90A,E129A (skp1dM) under the control of its own promoter from the single-copy vector *PSCNX* were transformed with the single-copy vector *RS316* expressing HA-tagged Gal80 under the control of the *ACT1* promoter. Cells were grown in glucose liquid media to OD_600 nm_ = 1 and induced with galactose liquid media for 1 h. Cycloheximide was added at time = 0 and the amount of Gal80 protein remaining in the cells after the indicated number of minutes was determined by Western blot with the help of an anti-HA antibody (upper panels). The membranes were stripped and stained with Coomassie Blue as loading controls (lower panels). (B) The ratio of the amount of HA-Gal80 protein to total protein (Coomassie) in *SKP1wt* cells (white bars) and *skp1dM* cells (black bars) for each time point in part A was determined with Image J. The ratio of the band intensities before the addition of cycloheximide (time = 0) was set as 1 and the error bars indicate the deviations between duplicates. (C) *JD52* cells expressing Nub-Skp1wt (line 1) and Nub-Skp1dM (lines 2 to 7) in place of endogenous Skp1 were 10-fold serially diluted, titrated onto the indicated plates, and incubated for 3 d on glucose plates and for 6 d on galactose plates containing 0.1 mg/l of the respiration inhibitor Antimycin A (AA). Cells over-expressed the indicated proteins from the multi-copy vector *YEplac112* under the control of their own respective promoters. Cells in line 7 expressed Skp1 from the single-copy vector *YCplac22* under the control of its own promoter. (D) *JD52* cells of the indicated genotype were grown in glucose liquid media to OD_600 nm_ = 1 and induced with galactose liquid media for 8 h. Cells over-expressed Gal3 from *RS314* under the control of the *ACT1* promoter or lacked *GAL80* as indicated. Total RNA was isolated and *GAL1* mRNA was determined relative to *ACT1* mRNA by quantitative real-time PCR. The value determined for *SKP1wt* cells grown with glucose liquid media was set as 1 and the error bars indicate the standard deviations between three replicates.

E3 ubiquitin ligases add ubiquitin to proteins that are targeted for degradation by the 26S proteasome [18], and Skp1 is an essential component of all SCF E3 ubiquitin ligases [19]. Previously, we had found that the Skp1 derivative Nub-HA-Skp1V90A,E129A (Skp1dM) causes the *gal* ^−^ phenotype when expressed in place of endogenous Skp1 [37]. We had isolated α2 as a multi-copy suppressor and shown that galactose-induced protein degradation of the repressor Mig2, which—like α2 [38]—uses the co-repressor Tup1, was abolished in the *skp1dM* strain [37]. The most likely explanation was that over-expression of α2 had titrated Tup1 away from *GAL1* promoter-bound Mig2, which—like Mig1 [39]—activated transcription in the absence of Tup1. The additional gene deletion of *MIG2*, however, had only partially suppressed the *gal* ^−^ phenotype of the *skp1dM* strain [37], suggesting that Skp1 mediated the galactose-induced protein degradation of additional transcription factors. Therefore, we wanted to see if Gal80 was a functionally relevant target of SCF E3 ubiquitin ligases. HA-Gal80 protein was degraded in galactose-induced *SKP1* wild-type cells (Figure 2A, lanes 5 to 8; Figure 2B, white bars), while it was stable in galactose-induced *skp1dM* cells (Figure 2A, lanes 13 to 16; Figure 2B, black bars), indicating that wild-type Skp1 was required for the galactose-induced protein degradation of Gal80. We transformed the *skp1dM* mutant strain with multi-copy plasmids expressing Sgt1 (which is required for Skp1-dependent cyclin degradation [40]), α2, Ubp3 (which dosage-compensates the *gal* ^−^ phenotype of cells expressing the proteolytically instable Tbp1E186D [41]), and Gal3. The over-expression of Sgt1 suppressed the temperature sensitivity of the *skp1dM* strain (Figure 2C, line 3). The over-expression of Gal3 and α2 suppressed the *gal* ^−^ phenotype, but not the temperature sensitivity, of the *skp1dM* mutant strain (Figure 2C, lines 4 and 6), while the over-expression of Ubp3 had no effect (Figure 2C, line 5). Real-time PCR quantification revealed that galactose induction of *GAL1* mRNA relative to *ACT1* mRNA was abolished in the *skp1dM* mutant strain (Figure 2D) and that it was restored to some 550-fold in the presence of excess Gal3 and almost fully in the absence of Gal80 (Figure 2D), providing genetic evidence that galactose-stable Gal80 had been the main cause for the *gal* ^−^ phenotype of the *skp1dM* strain.

### SCF^Mdm30^ Targets Gal80

**Figure 3:**
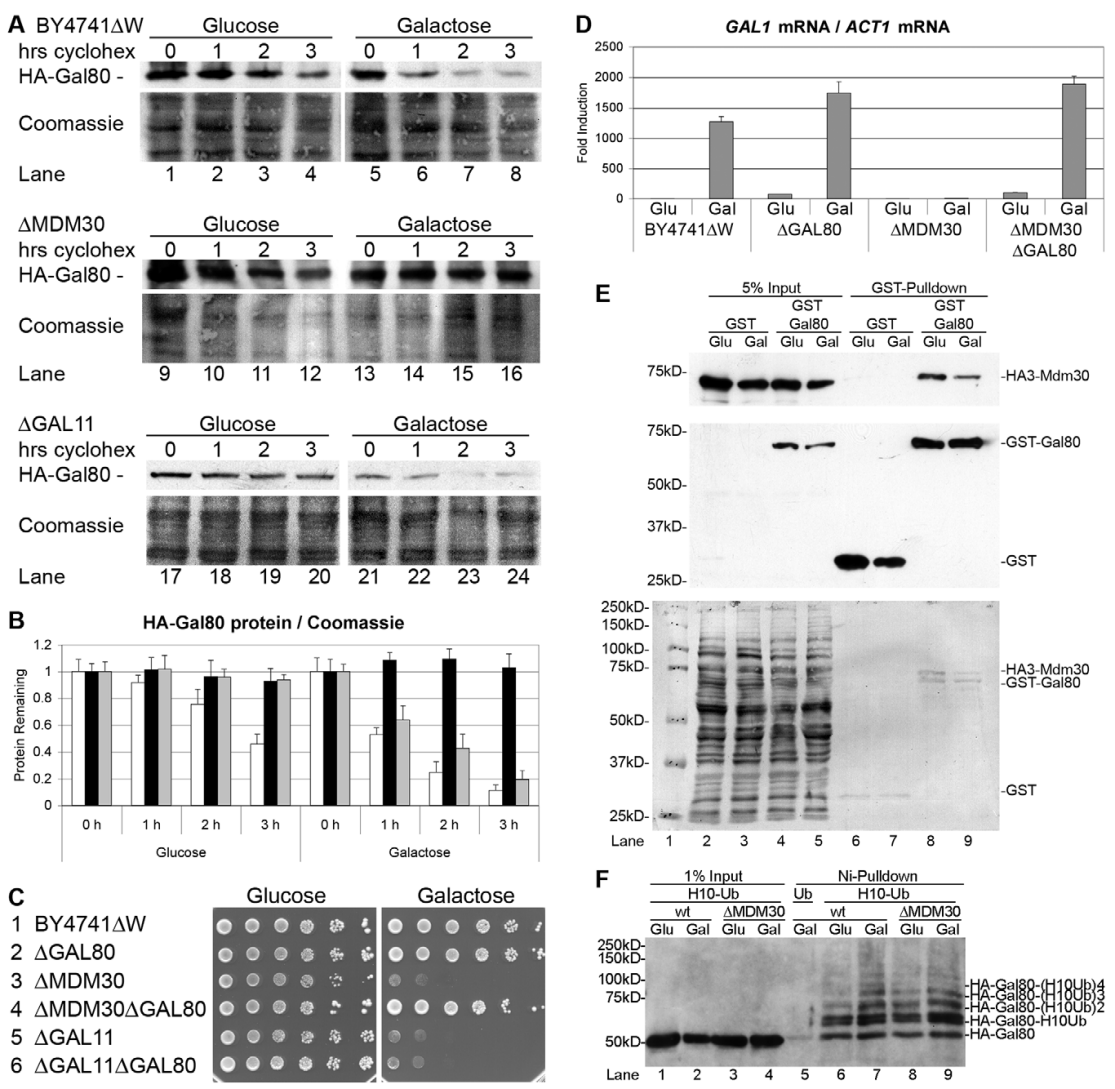
Gal80 is the main target of Mdm30. (A) HA-tagged Gal80 was expressed in *BY4741ΔW* cells of the indicated genotype. Cells were grown in glucose liquid media to OD_600 nm_ = 1 and induced with galactose liquid media for 1 h. Cycloheximide was added at time = 0 and the amount of Gal80 protein remaining in the cells after the indicated number of hours was determined by Western blot with the help of an anti-HA antibody (upper panels). The membranes were stripped and stained with Coomassie Blue as loading controls (lower panels). (B) The ratio of the amount of HA-Gal80 protein to total protein (Coomassie) in *BY4741ΔW* cells (white bars), *BY4741ΔWΔMDM30* cells (black bars), and *BY4741ΔWΔGAL11* cells (grey bars) for each time point in part A was determined with Image J. The ratio of the band intensities before the addition of cycloheximide (time = 0) was set as 1 and the error bars indicate the deviations between duplicates. (C) *BY4741ΔW* cells of the indicated genotype were 10-fold serially diluted, titrated onto the indicated plates, and incubated for 3 d on the glucose plate and for 6 d on the galactose plate. (D) *BY4741ΔW* cells of the indicated genotype were grown in glucose liquid media to OD_600 nm_ = 1 (Glu) and induced with galactose liquid media for 8 h (Gal). Total RNA was isolated and *GAL1* mRNA was determined relative to *ACT1* mRNA by quantitative real-time PCR. The value determined for *BY4741ΔW* wild-type cells grown with glucose liquid media was set as 1 and the error bars indicate the standard deviations between three replicates. (E) HA3-tagged Mdm30 and GST or GST-Gal80 were co-expressed in *BY4741ΔW* cells under the control of the *ACT1* promoter from the multi-copy vectors *RS423* and *RS424*, respectively. Cells were grown with glucose liquid media to OD_600 nm_ = 1 (odd lanes) and induced in galactose liquid media for 1 h (even lanes). GST and GST-Gal80 were pulled down from cell extracts with the help of glutathione beads, and Inputs and GST Pulldowns were analyzed by Western blots with the help of an anti-HA antibody (upper panel) and an anti-GST antibody (middle panel). The membrane was stripped and stained with Coomassie in order to compare the amount of protein loaded for Input and GST Pulldown (bottom panel). (F) Histidine-tagged ubiquitinated proteins were precipitated with Ni-beads from extracts of glucose-grown (Glu) and galactose-induced (Gal) *SUB288ΔWL* (lanes 1, 2, 5, 6, 7) and *SUB288ΔWLΔMDM30* (lanes 3, 4, 8, 9) cells expressing HA-Gal80 and ubiquitin (lane 5) or histidine-tagged ubiquitin (lanes 1 to 4 and 6 to 9) in place of endogenous ubiquitin. Inputs (lanes 1 to 4) and precipitates (lanes 5 to 9) were analyzed by Western blot with the help of an anti-HA antibody. The ubiquitinated bands appear as doublets, indicating that more than one lysine in Gal80 is subject to ubiquitination.

F-box proteins provide the substrate specificity to SCF E3 ubiquitin ligases [19], and the deletion of the gene encoding the F-box protein Mdm30 causes a *gal* ^−^ phenotype [20]. Cycloheximide chase assays demonstrated that HA-Gal80 was degraded in galactose-induced *BY4741ΔW* wild-type cells (Figure 3A, lines 5 to 8; Figure 3B, white bars), while it was stable in galactose-induced *ΔMDM30* cells (Figure 3A, lines 13 to 16; Figure 3B, black bars), suggesting that SCF^Mdm30^ targets Gal80 for galactose-induced protein degradation. Importantly, and consistent with a recent report [42], the additional gene deletion of *GAL80* suppressed the *gal* ^−^ phenotype of the *ΔMDM30* strain (Figure 3C, line 4). Gal80 was still degraded in galactose-induced *ΔGAL11* cells (Figure 3A, lanes 21 to 24; Figure 3B, grey bars) and the additional gene deletion of *GAL80* did not suppress the *gal* ^−^ phenotype of cells lacking Gal11 (Figure 3C, line 6), confirming that the suppression of the *gal* ^−^ phenotype of the *ΔMDM30* strain by the additional gene deletion of *GAL80* was gene-specific and that the F-box protein Mdm30 acts genetically upstream of the repressor Gal80, while the Mediator component Gal11 (Med15; which is a target of Gal4 [43]) acts genetically downstream of the repressor Gal80. Real-time PCR quantification of *GAL1* mRNA relative to *ACT1* mRNA confirmed that the additional gene deletion of *GAL80* fully suppressed the transcriptional defect of the *ΔMDM30* strain (Figure 3D), suggesting that Mdm30 targets mainly Gal80 for galactose-induced protein degradation. Consistently, GST-Gal80, but not GST, pulled down HA-tagged Mdm30 and Skp1 from yeast extracts (Figure 3E, lanes 8 and 9; Figure S6). Coomassie staining demonstrated that Gal80 and Mdm30 interacted at approximately equal amounts (Figure 3E, lanes 8 and 9). However, Gal80 interacted with Mdm30 (and Skp1) not only in galactose-induced but also in glucose-grown cells (Figure 3D, compare lanes 8 and 9; Figure S6), possibly reflecting the (slower) protein degradation of Gal80 in cells grown with glucose (Figure 3A, lane 4; Figure 3B, white bars). The half-life of Gal80 was calculated to be approximately 3 h in glucose-grown *BY4741ΔW* cells and approximately 1 h in galactose-induced *BY4741ΔW* cells. Gal80 had been completely stable in glucose-grown *H_10_Ub* cells (Figure 1, lines 1 to 4), indicating that the N-terminal tail of 10 histidines might have interfered with the slow protein degradation of Gal80 in glucose-grown cells. In agreement with the hypothesis that Gal80 is not just degraded in galactose-induced but also in glucose-grown cells (albeit with slower kinetics), Gal80 was poly-ubiquitinated in cells grown with glucose and in cells induced with galactose (Figure 3F, lanes 6 and 7). The amount of poly-ubiquitinated species of Gal80 was only slightly higher in galactose-induced cells as compared to in glucose-grown cells, suggesting that the generation of the poly-ubiquitinated species of Gal80 is rate-limiting, and once generated, poly-ubiquitinated Gal80 is immediately degraded. The ubiquitinated forms of HA-Gal80 are not visible in the input lanes, indicating that only a very small fraction of the Gal80 inside the cell is ubiquitinated at any point in time. The figure further shows that Gal80 was poly-ubiquitinated in wild-type cells as well as in cells lacking Mdm30 (Figure 3F, compare lanes 7 and 9), indicating that Mdm30 is not the only F-box protein targeting Gal80. In order to identify additional SCF E3 ubiquitin ligases targeting Gal80, we tested galactose utilization defective F-box protein gene deletion mutant strains [37] and found that Gal80 was also stable in galactose-induced cells lacking the F-box proteins Das1 and Ufo1 (Figure S7A,B). Importantly, the *gal* ^−^ phenotype of cells lacking Das1 and Ufo1 was suppressed by the additional gene deletion of *GAL80* (Figure S7C) and GST-Gal80, but not GST, pulled down Das1, and Ufo1 from yeast extracts (Figure S7D,E), indicating that targeting of Gal80 by at least these three F-box proteins is required for the efficient galactose-induced protein degradation of Gal80. Gal80 interacted with all three F-box proteins in cells grown with glucose and in cells grown with galactose. Consistently, the deletion of *MDM30*, *DAS1*, and *UFO1* stabilized Gal80 also in glucose-grown cells (Figures 3B and S7B). The signal observed for the pulldown of the F-box proteins with GST-Gal80 was higher in glucose-grown cells than in galactose-induced cells (compare lanes 8 and 9 in Figures 3E and S7D,E). A possible explanation is that in galactose-induced cells, more than in glucose-grown cells, the protein-protein interaction between the F-box proteins and Gal80 resulted in the protein degradation of Gal80, which means that the amount of the F-box protein pulled by GST-Gal80 does not necessarily reflect the strength of the protein-protein interaction. The over-expression of Mdm30 and Ufo1 suppressed the *gal* ^−^ phenotype of cells lacking Das1 (Figure S7F, lanes 3 and 4), indicating that galactose induction requires a critical threshold of Gal80-targeting SCF E3 ubiquitin ligases.

### Stable Derivatives of Gal80 Inhibit Galactose Induction

**Figure 4:**
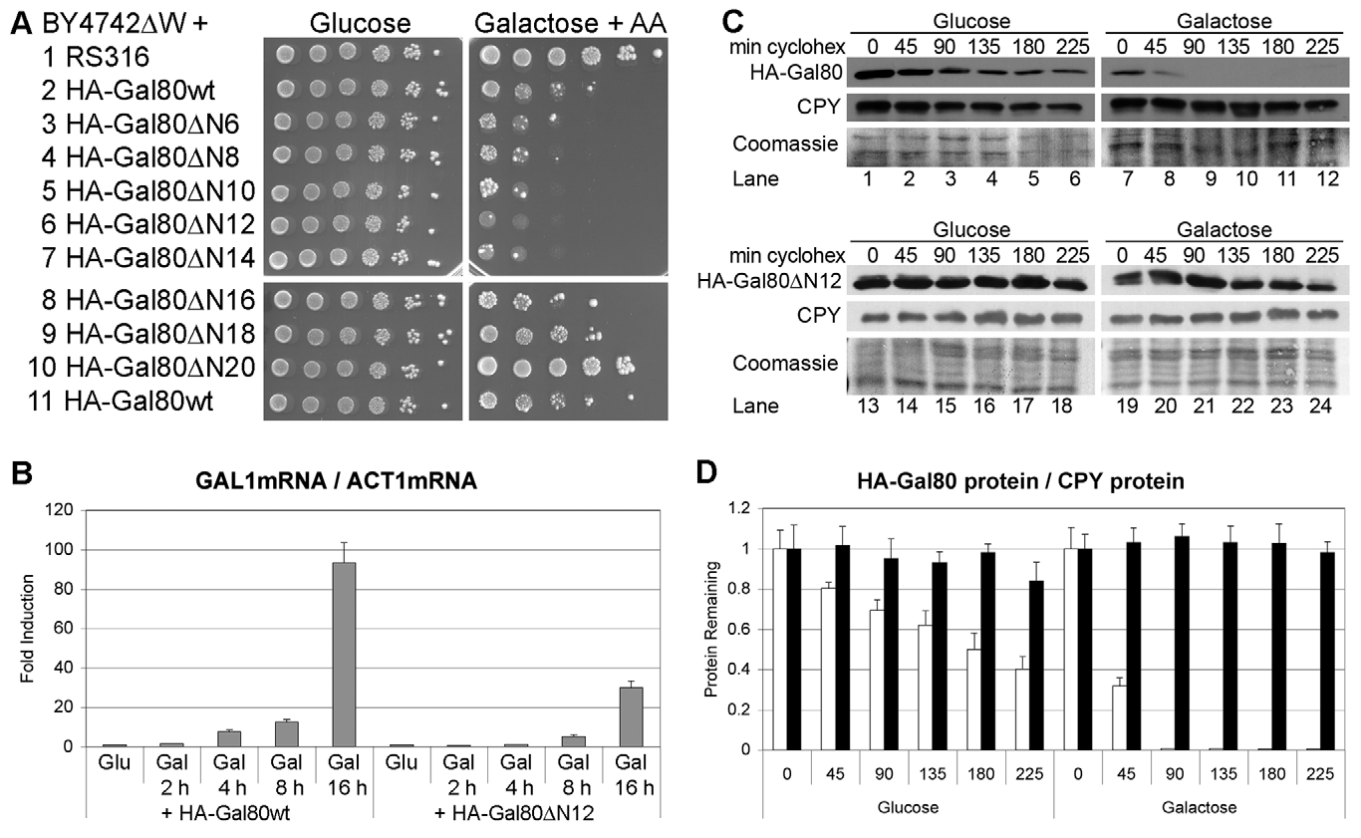
A galactose-stable Gal80 deletion derivative inhibits galactose induction of the *GAL1* gene. (A) *BY4742ΔW* cells over-expressing the indicated HA-Gal80 deletion derivatives from *RS316* under the control of the *ACT1* promoter were 10-fold serially diluted, titrated onto the indicated plates, and incubated for 3 d (Glucose) and 6 d (Galactose+AA = 1 mg/l Antimycin A), respectively. (B) Cells of part A, lines 1, 2 and 6, were grown in glucose liquid media to OD_600 nm_ = 1 and induced with galactose liquid media for the indicated number of hours. Total RNA was isolated and *GAL1* mRNA was determined relative to *ACT1* mRNA by quantitative real-time PCR. The value determined for *BY4742ΔW* cells containing *RS316* grown with glucose liquid media was set as 1 and the error bars indicate the standard deviations between three replicates. (C) *BY4742ΔW* cells expressing HA-tagged wild-type Gal80 or Gal80ΔN12 from *RS317* under the control of the *ACT1* promoter were grown in glucose liquid media to OD_600 nm_ = 1 and induced with galactose liquid media for 1 h. Cycloheximide was added at time = 0 and the amount of HA-Gal80 and HA-Gal80ΔN12 proteins remaining in the cells after the indicated number of minutes was determined by Western blot with the help of an anti-HA antibody (upper panels). The membranes were stripped and reprobed with an anti-CPY antibody (middle panels), followed by a second stripping and staining with Coomassie Blue as loading controls (lower panels). (D) The ratio of the amount of HA-Gal80 protein to CPY protein (white bars) and of HA-Gal80ΔN12 protein to CPY protein (black bars) in *BY4742ΔW* cells for each time point in part C was determined with Image J. The ratio of the band intensities before the addition of cycloheximide (time = 0) was set as 1 and the error bars indicate the deviations between duplicates.

SCF E3 ubiquitin ligases are enzymes that not only target Gal80 for ubiquitin proteasome-mediated protein degradation but also other proteins like Gal4 [20] and Mig2 [37]. It could be argued that defects in the protein degradation of some protein other than Gal80 had caused the *gal* ^−^ phenotype of the *H_10_UbD58A*, *skp1dM*, and *ΔMDM30* mutant strains. We have shown that the additional gene deletion of *GAL80* suppressed the transcriptional defects of all of these mutants, indicating that Gal80 is the only functionally relevant target, but in order to gain independent evidence that the galactose-induced protein degradation of Gal80 is required for the galactose induction of the *GAL* genes, we sought to generate a galactose-stable Gal80 derivative that would interfere with transcriptional activation of the *GAL* genes. Some degraded proteins contain an N-terminal degron, and we performed a series of small N-terminal deletions of Gal80 and tested them for causing defects in galactose utilization. The over-expression of wild-type HA-Gal80 reduced growth on a galactose plate in the presence of the respiration inhibitor Antimycin A (Figure 4A, line 2). The successive deletion of two amino acids increased the growth inhibition, with the deletion derivative lacking the 12 N-terminal amino acids of Gal80 showing the biggest growth inhibition (Figure 4A, line 6). N-terminal deletions of more than 12 amino acids resulted in less inhibition, with the Gal80 deletion derivative lacking the N-terminal 20 amino acids (which removes the first four residues of the Rossmann-fold [44]) having lost the ability to inhibit growth on the galactose plate (Figure 4A, line 10). Real-time PCR quantification of *GAL1* mRNA relative to *ACT1* mRNA showed that the over-expression of the HA-Gal80 derivative lacking the N-terminal 12 amino acids reduced galactose induction of the *GAL1* gene 5- to 3-fold more than the over-expression of wild-type HA-Gal80 (Figure 4B). Cycloheximide chase assays demonstrated that the HA-Gal80 deletion derivative lacking the N-terminal 12 amino acids was indeed stable in galactose-grown cells (Figure 4C, lanes 19 to 24; Figure 4D, black bars), confirming our hypothesis that galactose induction of the *GAL1* gene requires protein degradation of the repressor Gal80.

### Mediator Interacts with SCF E3 Ubiquitin Ligases

The essential Mediator subunit Srb7 (Med21) plays a pivotal role in the regulation of transcription [45],[46]. In order to identify human proteins interacting with the human Mediator component hSrb7, we fused it to the C-terminal half of ubiquitin that was extended by the RUra3 reporter (Cub-RUra3) and performed a Split-Ubiquitin screen [47],[48] with an expression library of human cDNAs fused to the N-terminal half of ubiquitin (Nub; Figure S8A). The Nub fusion of the human SCF E3 ubiquitin ligase component hSkp1 was isolated by its ability to confer FOA resistance to *S. cerevisiae* cells expressing hSrb7-Cub-RUra3 (Figure S8B), indicating that both proteins interacted inside the yeast cells. *E. coli*–expressed GST-hSrb7, but not GST, pulled down Nub-HA-hSkp1 from yeast extract (Figure S8C, lane 6), and *E. coli*–expressed GST-hSkp1, but not GST, pulled down *E. coli*–expressed H6-HA-hSrb7 (Figure S8C, lane 3), demonstrating that both proteins interacted directly with each other also in vitro. The human Split-Ubiquitin system (Figure S8D; [49]) was used to demonstrate that both proteins interacted with each other also in vivo (Figure S8E). hSrb7 and hSkp1 are subunits of distinct protein complexes, but the SCF component hSkp1 might play an additional role as a component of Mediator, while the Mediator component hSrb7 might moonlight as a component in an SCF complex. In order to distinguish between these possibilities, we performed co-immunoprecipitations with HeLa extracts and found that hSkp1 pulled down other Mediator components like hMed6 (Figure S9A, lane 3), while hMed6 pulled down other SCF components like hCul1 (Figure S9A, lane 10), indicating that hSrb7 and hSkp1 interacted with each other as components of their own respective complexes. We knocked down hSrb7 and hSkp1 in HeLa cells by RNA interference (Figure S9B), which dramatically reduced the heat-shock induction of the human *HSP70B'* gene (Figure S9C), indicating that hSrb7 and hSkp1 are functionally relevant for transcription in human cells. Skp1 is a component of the SCF E3 ubiquitin ligases, suggesting that protein degradation could be an important aspect of how Srb7 regulates transcription.

### Mediator Controls Gal80 Degradation

**Figure 5:**
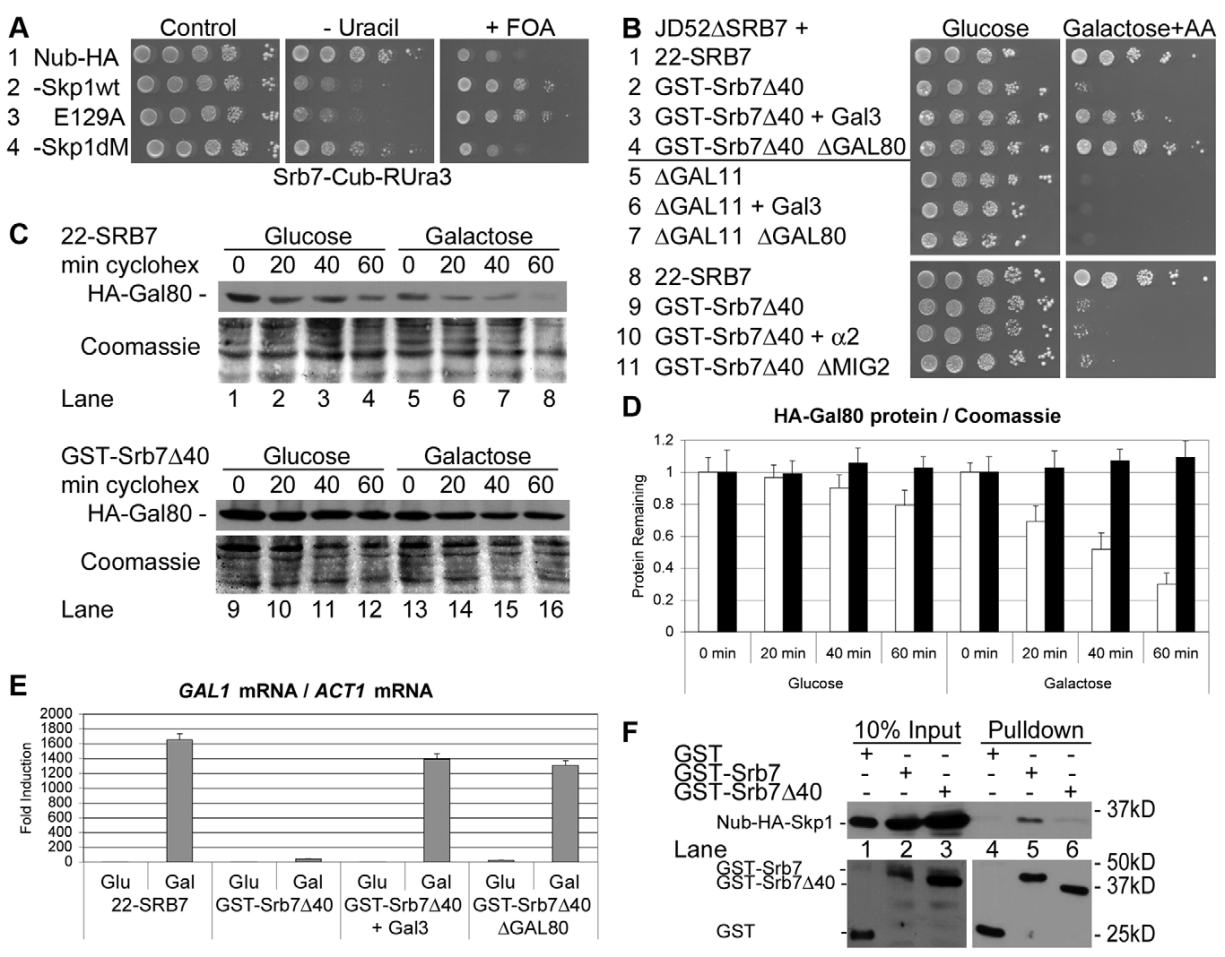
Mediator controls galactose-induced Gal80 degradation. (A) *JD52* cells expressing Srb7 fused to Cub-RUra3 and Nub-HA fused to the indicated derivatives of Skp1 were titrated onto the depicted plates and incubated for 3 d (Skp1dM = Skp1V90A,E129A). Protein-protein interaction between Srb7 and Skp1 is indicated by lack of growth on the uracil-depleted plate and by growth on the FOA-containing plate. (B) *JD52* cells whose chromosomal *SRB7* gene had been replaced by *HIS3* and that expressed wild-type Srb7 under the control of its own promoter from the single-copy vector *YCplac22* (22-SRB7; lines 1 and 8) or GST-Srb7 lacking the first 40 amino acids of Srb7 from the multi-copy vector *YG1u* under the control of the *ADH1* promoter (GST-Srb7Δ40; lines 2 to 4 and lines 9 to 11) and *BY4741ΔW* cells of the indicated genotype (lines 5 to 7) were 10-fold serially diluted, titrated onto the depicted plates, and incubated for 3 d on the glucose plate and for 6 d on the galactose plate containing 0.1 mg/l AA. Cells in lines 3 and 6 over-expressed Gal3 from *RS315* under the control of the *ACT1* promoter, cells in line 10 over-expressed α2 from *RS315* under the control of the *ACT1* promoter, cells in lines 4 and 7 lacked *GAL80*, and cells in line 11 lacked *MIG2*. (C) *JD52* cells whose chromosomal *SRB7* gene had been replaced by *HIS3* and that expressed wild-type Srb7 under the control of its own promoter from the single-copy vector *YCplac22* (22-SRB7) or GST-Srb7 lacking the first 40 amino acids of Srb7 from the multi-copy vector *YG1u* under the control of the *ADH1* promoter (GST-Srb7Δ40) were transformed with the single-copy vector *RS316* expressing HA-tagged Gal80 under the control of the *ACT1* promoter. Cells were grown in glucose liquid media to OD_600 nm_ = 1 and induced with galactose liquid media for 1 h. Cycloheximide was added at time = 0 and the amount of Gal80 protein remaining in the cells after the indicated number of minutes was determined by Western blot with the help of an anti-HA antibody (upper panels). The membranes were stripped and stained with Coomassie Blue as loading controls (lower panels). (D) The ratio of the amount of HA-Gal80 protein to total protein (Coomassie) in *22-SRB7* cells and *GST-Srb7Δ40* cells for each time point in part A was determined with Image J. The ratio of the band intensities before the addition of cycloheximide (time = 0) was set as 1 and the error bars indicate the deviations between duplicates. (E) Cells of part B, lines 1 to 4, were grown in glucose liquid media to OD_600 nm_ = 1 and induced with galactose liquid media for 8 h. Total RNA was isolated and *GAL1* mRNA was determined relative to *ACT1* mRNA by quantitative real-time PCR. The value determined for *22-SRB7* cells grown with glucose liquid media was set as 1 and the error bars indicate the standard deviations between three replicates. (F) Whole-cell extracts of *JD52* cells expressing the indicated Srb7 derivatives in place of endogenous Srb7 and Nub-HA-Skp1 were incubated with glutathione beads, and precipitates were washed five times. Inputs and precipitates were analyzed by Western blot with an anti-HA antibody (upper panel) and with an anti-GST antibody (lower panels).

The Split-Ubiquitin assay revealed that also the *S. cerevisiae* Srb7 and Skp1 proteins interacted with each other in vivo (Figure 5A, line 2). Interestingly, Skp1dM was defective for the protein interaction with Srb7 (Figure 5A, line 4). Our results showed that the Mediator of transcription interacts with SCF E3 ubiquitin ligases, and in order to see if Mediator plays a role in the galactose-induced protein degradation of Gal80, we generated a *gal* ^−^ allele of *SRB7* by replacing endogenous Srb7 with a GST fusion to a C-terminal fragment of Srb7 lacking the first 40 amino acid residues (Figure 5B, line 2). The over-expression of Gal3 and the deletion of *GAL80* suppressed the *gal* ^−^ phenotype of the *GST-Srb7Δ40* strain (Figure 5B, compare lines 1 to 4), indicating that excess Gal80 could have caused the *gal* ^−^ phenotype. The over-expression of Gal3 and the deletion of *GAL80* did not suppress the *gal* ^−^ phenotype of cells lacking the Mediator subunit Gal11 (Figure 5B, compare lines 5 to 7), demonstrating that the suppression had been gene-specific and that the Mediator subunit Srb7 acts genetically upstream of Gal80, while the Mediator subunit Gal11 acts genetically downstream of Gal80. The over-expression of α2 and the deletion of *MIG2* did not suppress the *gal* ^−^ phenotype of the *GST-Srb7Δ40* strain (Figure 5B, compare lines 8 to 11), while the over-expression of α2 and the deletion of *MIG2* had suppressed (partially) the *gal* ^−^ phenotype of the *skp1dM* strain (Figure 2C, line 4 and [37]), suggesting that Skp1 acts genetically upstream of both Gal80 and Mig2, while Srb7 acts genetically upstream of Gal80 only. Cycloheximide chase assays demonstrated that Gal80 was degraded in galactose-induced cells expressing wild-type Srb7 (Figure 5C, lanes 5 to 8; Figure 5D, white bars), but stable in galactose-induced *GST-Srb7Δ40* cells (Figure 5C, lanes 13 to 16; Figure 5D, black bars), indicating that Mediator controls the galactose-induced protein degradation of Gal80. Real-time PCR quantification confirmed that galactose induction of *GAL1* mRNA relative to *ACT1* mRNA was abolished in the *GST-Srb7Δ40* strain and that it was almost fully restored by the over-expression of Gal3 and the deletion of *GAL80* (Figure 5E), suggesting that the failure of the *GST-Srb7Δ40* strain to degrade Gal80 upon galactose induction had been the main cause for the failure to activate the transcription of the *GAL1* gene. GST-Srb7, but not GST, pulled down Skp1 from yeast extract, while GST-Srb7Δ40 failed to do so (Figure 5F, lanes 5 and 6), indicating that the protein-protein interaction with Skp1 is mediated by the N-terminus of Srb7, which is the most conserved part of the protein [46]. Our results have shown that the degradation of Gal80 was abolished when endogenous Skp1 was replaced by a mutant Skp1 derivative that failed to interact with Srb7 and when endogenous Srb7 was replaced by a Srb7 mutant protein that failed to interact with Skp1, suggesting that the protein-protein interaction between the Mediator component Srb7 and the SCF component Skp1 is required for the protein degradation of Gal80.

### Snf1 Directs Galactose-Induced Gal80 Degradation

**Figure 6:**
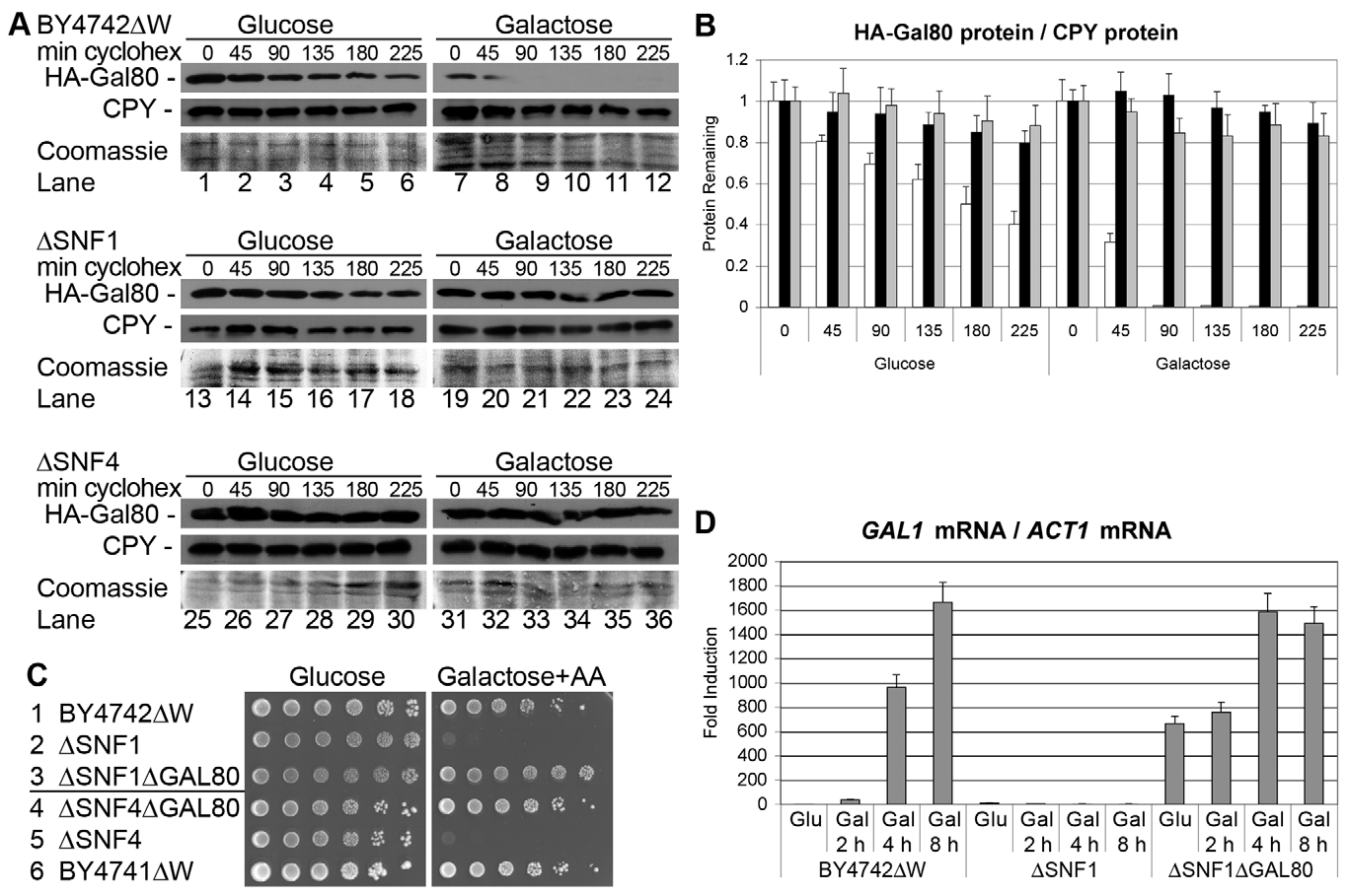
The protein kinase Snf1 is required for galactose-induced protein degradation of Gal80. (A) *BY4742ΔW* (lanes 1 to 12), *BY4742ΔWΔSNF1* (lanes 13 to 24), and *BY4741ΔWΔSNF4* (lanes 25 to 36) cells expressing HA-tagged Gal80 under the control of the *ACT1* promoter were grown in glucose liquid media to OD_600 nm_ = 1 and induced with galactose liquid media for 1 h. Cycloheximide was added at time = 0 and the amount of Gal80 protein remaining in the cells after the indicated number of minutes was determined by Western blot with the help of an anti-HA antibody (upper panels). The membranes were stripped and reprobed with an anti-CPY antibody (middle panels), followed by a second stripping and staining with Coomassie Blue as loading controls (lower panels). (B) The ratio of the amount of HA-Gal80 protein to CPY protein in *BY4742ΔW* cells (white bars), *BY4742ΔWΔSNF1* cells (black bars), and *BY4741ΔWΔSNF4* cells (grey bars) for each time point in part A was determined with Image J. The ratio of the band intensities before the addition of cycloheximide (time = 0) was set as 1 and the error bars indicate the deviations between duplicates. (C) *BY4742ΔW* cells (lines 1 to 3) and *BY4741ΔW* cells (lines 4 to 6) of the indicated genotype were 10-fold serially diluted, dropped onto the depicted plates, and incubated for 6 d at 28°C. The galactose plate contained 1 mg/l Antimycin A. (D) Cells of part C, lines 1 to 3, were grown in glucose liquid media to OD_600 nm_ = 1 and induced with galactose liquid media for the indicated number of hours. Total RNA was isolated and *GAL1* mRNA was determined relative to *ACT1* mRNA by quantitative real-time PCR. The value determined for *BY4742ΔW* cells grown with glucose liquid media was set as 1 and the error bars indicate the standard deviations between three replicates.

Mediator acts upstream of the activator Gal4 by controlling the galactose-induced protein degradation of the inhibitor Gal80. But how does Mediator know about the switch in carbon source? The protein kinase Snf1 is required for the transcription of glucose-repressed genes in *S. cerevisiae*, and the deletion of *SNF1* resulted in the failure to degrade Gal80 (Figure 6A, lanes 19 to 24; Figure 6B, grey bars), to utilize galactose (Figure 6C), and to activate the *GAL1* gene under inducing conditions (Figure 6D). The activating gamma subunit Snf4 is required for the kinase activity of the SNF1 complex and Gal80 was also stable in galactose-induced *ΔSNF4* cells (Figure 6A, lanes 31 to 36; Figure 6B, grey bars), indicating that the kinase activity of the SNF1 complex is required for the degradation of Gal80. The additional gene deletion of *GAL80* fully suppressed the transcriptional defect of *ΔSNF1* and *ΔSNF4* cells (Figure 6C, lines 3 and 4; Figure 6D), but no interaction was observed between Snf1 and Gal80 in a pulldown assay (Figure S10), indicating that Snf1 controls *GAL1* expression mainly by targeting Gal80 via Srb7 and SCF E3 ubiquitin ligases. The Split-Ubiquitin assay did not reveal an interaction between Snf1 and Srb7 (Figure S11, line 23), however Srb7 is a component of Mediator and Snf1 interacted with the Mediator components Med6 (Figure S11, line 6), Med10 (Figure S11, line 11), Srb6 (Med22; Figure S11, line 21), and Srb11 (CycC; 11, line 27). The protein interaction between the kinase Snf1 and the Mediator component Srb11 had been observed both in vivo and in vitro previously [50],[51]. Srb11 is a cyclin-like cofactor for the protein kinase Srb10 (Cdk8), and the Mediator components Srb10 and Srb11 are both required for the full transcriptional activation of the *GAL1* gene [52]. Gal80 was stable in galactose-induced *ΔSRB10* and *ΔSRB11* cells (Figure S12A, lanes 7 to 12 and 19 to 24; Figure S12B), confirming that Snf1 might transduce the signal to degrade Gal80 via the Mediator subunit Srb11. The additional gene deletion of *GAL80* suppressed the galactose utilization defect of cells lacking Srb10 and Srb11 (Figure S12C, lines 3 and 5), providing genetic evidence that galactose-stable Gal80 had caused the *gal* ^−^ phenotype of *ΔSRB10* and *ΔSRB11* cells.

The SNF1 kinase is activated by the absence of glucose, but transcriptional activation of the *GAL* genes requires additionally the presence of galactose, as transcription of *GAL1* is not activated in cells grown with—for example—raffinose (Figure S13B). Consistently, Gal80 was more stable in cells grown with raffinose than in cells grown with galactose (Figure S14). The half-life of Gal80 in *BY4741ΔW* cells was calculated to be approximately 3 h when the cells were grown with glucose, 2 h when the cells were grown with raffinose, 1 h when galactose-induced cells had been pre-grown with glucose, and half an hour when the galactose-induced cells had been pre-grown with raffinose. However, our observations also indicate that active SNF1 kinase is necessary but not sufficient for the galactose-stimulated protein degradation of Gal80. An additional transducer that signals the presence of galactose is apparently required. A possible candidate for such a signal transducer is Gal3, as it is known to bind both galactose and Gal80 [14]. Cells lacking Gal3 display a strong *gal* ^−^ phenotype (Figure S15A, lines 3 and 4), which is suppressed by the additional gene deletion of *GAL80* (Figure S15A, lines 5 and 6), but the degradation of Gal80 in galactose-induced cells remained unchanged upon the deletion of *GAL3* (Figure S15B, lanes 5 to 8; Figure 15C), indicating that Gal3 does not play a role in the galactose-induced protein degradation of Gal80 and that galactose must utilize another transducer to stimulate the protein degradation of Gal80.

## Discussion

**Figure 7:**
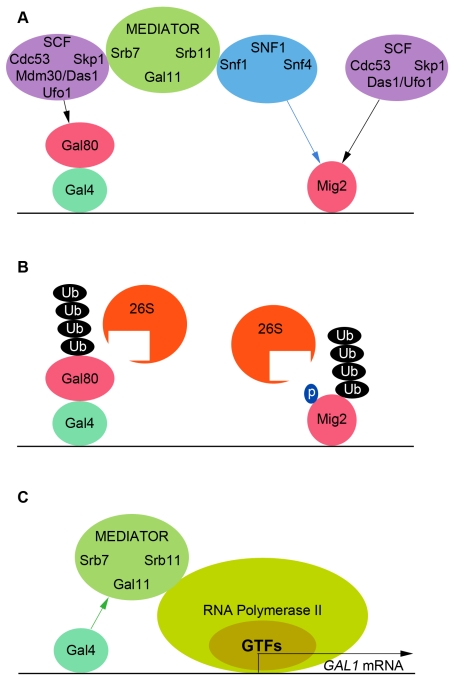
How Mediator orchestrates its own recruitment to the *GAL1* promoter. (A) The protein kinase SNF1 senses the absence of glucose and phosphorylates Mig2. Phosphorylated Mig2 is ubiquitinated by the E3 ubiquitin ligases SCF^Das1/Ufo1^ [37]. SNF1 also signals Mediator the absence of glucose via the Snf1-Srb11 interaction. Mediator transduces the signal to the E3 ubiquitin ligases SCF^Mdm30/Ufo1/Das1^ via the Srb7-Skp1 interaction. SCF^Mdm30/Ufo1/Das1^ ubiquitinate Gal80 via the interaction of Gal80 with the F-box proteins Mdm30, Ufo1, and Das1. (B) Poly-ubiquitinated Gal80 and Mig2 are degraded by the 26S proteasome. (C) Gal4 recruits chromatin-remodeling and chromatin-modifying complexes as well as the Holoenzyme of Transcription (consisting of RNA Polymerase II, the General Transcription Factors, and Mediator) to the *GAL1* promoter genes via the Gal4-Gal11 interaction [43].

Mediator was isolated by its ability to respond to transcriptional activators, and all studies published about Mediator have focused on the role of Mediator past its recruitment to the promoter by the activator [28]. Once recruited, Mediator is required to recruit the General Transcription Factors and RNA Polymerase II and to initiate transcription [29]. Mediator also affects post-initiation steps by affecting transcription elongation and chromatin structure [31]. We have shown here that Mediator additionally acts upstream of the activator Gal4 by controlling the degradation of the inhibitor Gal80. In cells grown with glucose, Gal80 binds to the activation domain of Gal4 and prevents it from activating transcription. Upon galactose induction, Mediator initiates the degradation of Gal80 via its interaction with the SCF E3 ubiquitin ligase component Skp1. Therefore, Mediator actually orchestrates its own recruitment to the *GAL1* promoter by regulating the activity of Gal4 (Figure 7).

SCF^Mdm30^ targets not only Gal80 but also Gal4 in galactose-induced cells, leading to the mono-ubiquitination and subsequent poly-ubiquitination and protein degradation of Gal4 [20]. In galactose-induced cells lacking Mdm30, Gal4 is no longer ubiquitinated and no longer degraded [20]. One could argue that changes in the proteolytic stability of Gal4 or in its mono-ubiquitination status might have been the cause for the *gal* ^−^ phenotypes that we have observed for the various *H_10_UbD58A*, *skp1*, *mdm30*, *srb7*, and *snf1* mutant strains described here. Therefore, it is important to note that our claim that the degradation of Gal80—and not the degradation of Gal4—is required for the transcriptional activation of the *GAL* genes is not just based on a simple correlation between the proteolytic stability of Gal80 and the inability of the cell to activate transcription of the *GAL1* gene, but on functional suppression. The additional gene deletion of *GAL80* fully suppressed the transcriptional defects of the *H_10_UbD58A*, *skp1*, *mdm30*, *srb7*, and *snf1* mutant strains. This means that in the absence of Gal80, Gal4 activated transcription in all these mutant strains just fine, which demonstrates that any effects that these strain mutations might have had on Gal4 were not relevant for Gal4's function as a transcriptional activator. Therefore, while mono-ubiquitination of Gal4 was certainly affected in the *H_10_UbD58A* strain (since endogenous wild-type ubiquitin had been replaced with H_10_UbD58A), Gal4-H_10_UbD58A fully activated transcription of the *GAL1* gene in the absence of Gal80, suggesting that H_10_UbD58A still protected Gal4 from the UAS-stripping activity of the 19S proteasome [25]. Furthermore, Gal4 fully activated transcription in cells lacking both Mdm30 and Gal80, which argues that Gal4 does not have to be degraded to become transcriptionally active. In addition, we have generated a galactose-stable Gal80 derivative that inhibited galactose induction in otherwise wild-type cells, which means that we have presented evidence for our claim that galactose induction requires Gal80 degradation that did not rely on a mutant strain background.

The deletion of the three F-box protein-coding genes *MDM30*, *DAS1*, and *UFO1* completely abolished galactose induction of *GAL1* mRNA (Figure 3D and [37]). Das1 and Ufo1 (but not Mdm30) also target the repressor Mig2 for galactose-induced protein degradation [37]. However, the additional gene deletion of *MIG2* did not increase galactose induction of *GAL1* mRNA in the *ΔUFO1* strain and had only a very small effect on the galactose induction of the *GAL1* mRNA in the *ΔDAS1* strain [37]. Therefore, an additional target for Das1 and Ufo1 had been proposed, and we have now shown here that Gal80 is this functionally relevant target, as Gal80—like Mig2 [37]—became stable in galactose-induced cells lacking Das1 and Ufo1 (Figure S7A and S7B), and the additional gene deletion of *GAL80* suppressed the *gal* ^−^ phenotype of both the *ΔDAS1* and the *ΔUFO1* strains (Figure S7C). We are proposing that a critical concentration of the three F-box proteins Mdm30, Das1, and Ufo1 is required for the galactose-stimulated protein degradation of Gal80. If any one of these three F-box proteins is missing, the concentration of the remaining two F-box proteins is insufficient for targeting of Gal80; Gal80 is not degraded and excess Gal80 prevents Gal4 from activating the *GAL* genes under inducing conditions. In support of this model (Figure 7), we were able to show that the *gal* ^−^ phenotype of *ΔDAS1* cells was suppressed by the over-expression of Ufo1 and Mdm30 (Figure S7E).

Gal3 sequesters Gal80 in the cytoplasm upon galactose induction [14]. The gene deletion of *GAL3* had caused a *gal* ^−^ phenotype that was suppressed by the additional gene deletion of *GAL80*, but the protein degradation of Gal80 was still stimulated in galactose-induced cells lacking Gal3 (Figure S15), indicating that instable Gal80 was not sufficient to allow Gal4 to activate transcription in the absence of Gal3. On the other hand, sequestration of Gal80 into the cytoplasm by endogenous levels of Gal3 was not sufficient to allow Gal4 to activate transcription in the presence of stable Gal80. Apparently, sequestration of Gal80 into the cytoplasm by Gal3 and ubiquitin-mediated protein degradation of Gal80 are both required for the galactose induction of the *GAL* genes.

Contrary to a previous report [20], we found that the deletion of the gene encoding the F-box protein Mdm30 abolished galactose induction of the *GAL1* mRNA. We have grown the cells in glucose liquid media prior to the switch to galactose liquid media—which is consistent with the switch in carbon sources conducted for the plate assay—while Muratani et al. grew the cells in raffinose liquid media prior to the switch to galactose liquid media. In order to determine if this difference in protocols was the cause for the difference in results, we performed the galactose induction with cells that had been pre-grown in raffinose, and we found that in this case, galactose-induced protein degradation of Gal80 and galactose induction of *GAL1* mRNA relative to *ACT1* mRNA were restored in the *ΔMDM30* strain (Figures S13B and S14). One other remarkable difference between the two growth protocols is the speed of induction. Galactose induction of *GAL1* mRNA relative to *ACT1* mRNA takes 4 h if the cells are pre-grown with glucose and only 1 h if the cells are pre-grown with raffinose (Figure S16). Consistently, cycloheximide chase assays demonstrate that Gal80 is more rapidly degraded in galactose-induced cells when the cells had been pre-grown with raffinose instead of with glucose (compare Figures 3B and S14B). The half-life of Gal80 in galactose-induced *BY4741ΔW* cells was approximately 1 h when the cells had been pre-grown with glucose and only half an hour when the cells had been pre-grown with raffinose. The correlation of the kinetics of galactose-induced Gal80 destruction and *GAL1* mRNA production suggests that the degradation of Gal80 is the rate-limiting step for the galactose induction of the *GAL1* gene.

## Materials and Methods

### Strains and Plasmids

The *S. cerevisiae* strain *SUB288* [53] has all chromosomal ubiquitin genes deleted and allows for the expression of plasmid-born ubiquitin derivatives in place of endogenous ubiquitin (see Table S1 for the genotypes of the strains and Table S2 for the sequences of PCR primers). However, the strain fails to grow on galactose plates containing the respiration inhibitor Antimycin A (AA). Transformation of the strain with single-copy vectors expressing Gal3 from its own promoter allowed the strain to grow on galactose AA plates and sequencing of the chromosomal *GAL3* gene demonstrated that *SUB288* carries a frame shift in the third codon of *GAL3*. The *TRP1* and *LEU2* genes were deleted and the defective *gal3* gene was repaired by homologous recombination with a wild-type *GAL3* PCR fragment followed by selection on a galactose AA plate or with *YIplac204-GAL3*, a derivative of the *TRP1*-marked integrative vector *YIplac204* [54] containing the *GAL3* gene, resulting in *SUB288GAL3ΔWL+316-Ub* and *SUB288GAL3ΔL+316-Ub*, respectively. DNA sequencing of PCR fragments derived from genomic DNA was used to confirm that the *GAL3* gene had been repaired. The ubiquitin point mutants were generated by two-step PCR with degenerate primers and cloned into the *LYS2*-marked single-copy vector *RS317* [55] containing the *ACT1* promoter-terminator cassette and into *RS317* expressing 10 histidines from the *ACT1* promoter. The *317-Ub and 317-H10-Ub* plasmids were transformed into *SUB288GAL3ΔWL+316-Ub* and *316-Ub* was shuffled out on FOA plates. All ubiquitin mutant strains were confirmed by DNA sequencing. The *GAL80* gene of *SUB288GAL3ΔWL+317-Ub* was knocked out with a derivative of *NKY51* [56], which carried the *hisG-URA3-hisG* cassette in the *Bgl*II site at nucleotide 612 of *GAL80*. *317-Ub* was replaced by *316-Ub* via plasmid loss, and plasmid shuffle was used to generate the *317-H10-Ub* and *317-H10-UbD58A* strains carrying *hisG* integrated into *GAL80*. The essential *SRB7* gene of *JD52* and *JD52ΔGAL80* was knocked out with a PCR fragment containing the *HIS3* gene flanked by 50 bp of *SRB7* promoter and terminator in the presence of *33-SRB7*, a derivative of the *URA3*-marked single-copy vector *YCplac33* [54] that expressed Srb7 from its own promoter. GST-Srb7 and GST-Srb7Δ40 were expressed from the *TRP1*-marked multi-copy vector YG1μ under the control of the *ADH1* promoter. *BY4741ΔW* and *BY4742ΔW* and their gene deletion derivatives were obtained from the respective EUROSCARF strains by inserting *hisG* into the *TRP1* gene with the help of *NKY1009* [56]. *YEp13-GAL3* was isolated from a *LEU2*-marked multi-copy *YEp13*-based genomic DNA library [37] as a multi-copy suppressor of the *gal* ^−^ phenotype of the *H_10_UbD58A* strain. *YEp13-GAL3* contains a 2,643 bp genomic DNA fragment with the entire *GAL3* gene, including 842 bp of promoter and 238 bp of terminator DNA. *112-GAL3* is a derivative of the *TRP1*-marked multi-copy vector *YEplac112* [54] containing the genomic *GAL3* fragment. *314-Gal3* is a derivative of the *TRP1*-marked single-copy vector *RS314* [55], expressing Gal3 from the *ACT1* promoter. *315-Gal3* is a derivative of the *LEU2*-marked single-copy vector *RS315* [55], expressing Gal3 from the *ACT1* promoter. *316-HA-Gal80* is a derivative of *RS316*, expressing Gal80 from the *ACT1* promoter. The N-terminal deletion derivatives of Gal80 were cloned into the same vector. *423-HA3-Mdm30*, *423-HA3-Das1*, and *423-HA3-Ufo1* are derivatives of the *HIS3*-marked multi-copy vector *RS423* [55], expressing Mdm30, Das1, and Ufo1 tagged with three HA epitopes from the *ACT1* promoter. *424-GST* and *424-GST-Gal80* are derivatives of the *TRP1*-marked multi-copy vector *RS424* [55], expressing GST and GST-Gal80 from the *ACT1* promoter. *YIplac128-Snf1c-HA_3_H_10_* is a derivative of the *LEU2*-marked integrative vector *YIplac128* [54], containing a C-terminal *Bgl*II-*Sal*I fragment of *SNF1* lacking the stop codon, and *YIplac128-Skp1c-HA_3_H_10_* is a derivative of *YIplac128* containing a C-terminal *Eco*RI-*Sal*I fragment of *SKP1* lacking the stop codon. Snf1-HA_3_H_10_ was expressed from the *SNF1* promoter following digestion with *Mlu*I and integration into the *SNF1* locus, while Skp1-HA_3_H_10_ was expressed from the *SKP1* promoter following digestion with *Ava*I and integration into the *SKP1* locus.

### Split-Ubiquitin Screen

A Clontech library derived from human B-cell cDNAs was partially digested with *Sau*3A and cloned into the *Bgl*II site of *PADNX-Nub-IBC* [57] in all three reading frames, resulting in 60,000 independent *DH5α* transformants. hSrb7 was cloned into *Pcup1-Cub-RUra314* [57] and transformed together with the Nub library into *JD52* [58], resulting in 160,000 transformants, which were plated onto FOA plates containing 10 µM CuSO_4_. The Nub plasmids from the 10 arising colonies were isolated and transformed back into *JD52* containing *hSrb7-Cub-Ura314*. Only one was plasmid-linked, and it contained the entire hSkp1 open reading frame fused to Nub-HA.

### mRNA Quantification

HeLa cells were grown to 80% confluency and transfected with 2 µg of pSuper (OligoEngine) construct and 5 µl of lipofectamin in serum-free DMEM for 5 h before being transferred into regular DMEM. The three constructs used were an empty vector as a negative control, siRNA specific for *hSKP1*, and siRNA specific for *hSRB7*. 48 h after transfection, one set of cells was heat-shocked at 45°C for 15 min and allowed to recover for 1 h in a 37°C incubator. A non-heat-shock sample was also incubated at 37°C for an identical length of time. These cells were then harvested by trypsinization and their mRNA was extracted using a Qiagen RNA Easy Kit. 300 nM of mRNA was utilized for reverse transcription primed by random hexamers, and the cDNA was quantified using Sybr-Green in an ABI Prism. Primers for *HSP70B'* mRNA were 5′-ccccatcattgaggaggttg-3′ and 5′-gaagcagaagaggatgaacc-3′. Primers for *hSKP1* mRNA were 5′-gcaaagagaaccagtggtgtga-3′ and 5′-aggtttgggatctgtgctcaa-3′. Primers for *hSRB7* mRNA were 5′-aatgtggtcctcctgcctctt-3′ and 5′-ccagaagcatgtctcctcgata-3′. Primers for *GAPDH* mRNA were 5′-ctctctgctcctcctgttcgac-3′ and 5′-tgagcgatgtggctcggct-3′.

*S. cerevisiae* cells were cultured in synthetic complete 2% (w/v) glucose medium at 28°C. At OD_600 nm_ = 1, the cells were collected by centrifugation. Galactose induction was performed by resuspending the cells in 2% galactose medium and incubation for the indicated amount of time. Total RNA was isolated using the RNAeasy Mini Kit (Qiagen) according to the manufacturer's protocol. cDNA was generated by reverse transcription PCR using Taqman MicroRNA Reverse Transcription Kit (Roche Applied Biosystems). Quantitative real-time PCR was performed using SYBR Green PCR Master Mix (Applied Biosystems). Primers used for *ACT1* mRNA were 5′-gaccaaactacttacaactcca-3′ and 5′-cattctttcggcaatacctg-3′. Primers used for *GAL1* mRNA were 5′-acttgcaccggaaaggtttg-3′ and 5′-ttggtacatcaccctcacagaaga-3′. All mRNA quantifications were performed three times, and the error bars represent the standard deviations.

### Coimmunoprecipitation

HeLa cells were grown to 80% confluency before they were transfected with 2 µg of *pCMV-myc-hSKP1* or *pCMV-myc* vector and 5 µl of lipofectamin in serum-free DMEM for 5 h before being transferred into regular DMEM. The cells were harvested 48 h after transfection by trypsinization and lysed in 1× PBS by freeze-thaw. The cell lysate was subsequently agitated on a rotor with 2 µl of anti-myc affixed agarose beads (Sigma) in 500 µl of ice cold 1× PBS overnight. The beads were washed four times with 1 ml PBS prior to heat elution at 95°C for 15 min. Proteins were separated on a 12% gel, transferred to a nitrocellulose membrane, which was probed with anti-Med6 rabbit polyclonal antibody (Abcam).

HeLa cells were grown to 80% confluency before they were harvested by trypsinization and lysed in 1× PBS by freeze-thaw. The cell lysate was diluted 1∶5 with RIPA buffer (50 mM Tris-HCl ph 8, 150 mM NaCl, 2 mM EDTA, 1% NP-40, 0.5% Sodium deoxycholate, 0.1% SDS) and incubated with 10 µl of rProtein G Sepharose (GE Healthcare) as well as 5 µl of anti-Med6 rabbit polyclonal antibody (Abcam) or anti-Cul1 mouse monoclonal antibody (Abcam) for 3 h. The sepharose was washed four times with 1 ml PBS and heat eluted at 95°C for 15 min. Proteins were separated on a 12% gel and transferred to a nitrocellulose membrane, which was probed with the reciprocal antibody (anti-Cul1 mouse monoclonal antibody (Abcam) or anti-Med6 rabbit polyclonal antibody (Abcam), respectively).

### GST Pulldown Assays

GST pulldown assays were performed using whole cell *S. cerevisiae* extracts prepared by bead beating in yeast lysis buffer (100 mM Tris pH 7.5, 50 mM KCl, 1 mM EDTA, 0.1% NP40) and whole cell *E. coli* extracts prepared by freeze-thaw in PBS (Phosphate-Buffered Saline). 500 µl of whole cell extract was added to equilibrated glutathione beads (Amersham Biosciences) containing 2 mM PMSF and 1 mM DTT. The reaction mixture was incubated at 4°C for 1 h. The sample was centrifuged at 3,000 rpm and the supernatant was removed. The glutathione beads were washed five times before Western Blot analysis.

### Ni Pulldown Assays

*S. cerevisiae* cells were grown in 50 ml synthetic complete 2% glucose medium to OD_600 nm_ = 1 and harvested by centrifugation. The cell pellets were suspended in 1 ml yeast breaking buffer (Triton X-100, 10% SDS, 5 M NaCl, 1 M Tris-Hcl pH 8, 0.5 M EDTA; Figure 5D) or yeast lysis buffer (Figure S6), pipetted into a screw-cap microcentrifuge tube containing acid-washed glass beads (Sigma-Aldrich, USA), and 2 mM PMSF was added. The tubes were then subjected to homogenization with a bead beater for 1 min and then rested on ice for 3 min. This process was repeated for three times. The samples were then centrifuged for 15 min at 13,000 rpm, and the supernatants were incubated with 10 µl of equilibrated nickel beads for 1 h at 4°C. After incubation, the samples were centrifuged at 3,000 rpm for 2 min. The nickel beads were washed with 1 ml yeast breaking/lysis buffer containing 20 mM imidazole. This washing process was repeated five times. The bound protein was eluted from the nickel beads using 100 µl of yeast breaking/lysis buffer with 500 mM imidazole for 30 min. This process was repeated two times. The supernatant was collected and stored at −80°C.

### Cycloheximide Chase Assays

*S. cerevisiae* cells were grown in liquid drop out media containing 2% glucose or raffinose to OD_600 nm_ = 1. Half of the cultures were induced in liquid media containing 2% galactose for 1 h before the addition of 200 mg/l cycloheximide (Sigma). Aliquots were taken at the indicated time points, and cellular proteins were analyzed by Western Blot with primary antibodies against hemagglutinin (HA; Roche) and carboxypeptidase Y (CPY; Molecular Probes), followed by staining with a horseradish peroxidase-coupled secondary anti-mouse IgG antibody and by Coomassie Brilliant Blue (Sigma) staining. The intensities of the bands were quantified with Image J (rsb.info.nih.gov/ij/index.html). The ratio of the band intensities before the addition of cycloheximide (time = 0) was set as 1, and the error bars represent the deviations between duplicates. Representative Western blots are shown. No significant differences were observed when the HA-Gal80 bands were normalized to CPY or to Coomassie staining. The half-life of Gal80 was calculated using trendline (excel).

## Supporting Information

Figure S1 Identification of galactose utilization-defective Ub mutants. Ten-fold serial dilutions of *SUB288GAL3ΔWL* cells expressing the indicated ubiquitin derivatives in place of endogenous ubiquitin were titrated onto the depicted plates and incubated at 28°C for 6 d. The ubiquitin derivatives were expressed from the *LYS2*-marked single-copy vector *RS317* under the control of the *ACT1* promoter. The galactose plates contained 1 mg/l of the respiration inhibitor Antimycin A (AA).
(TIF)

Figure S2 Summary of the results of the alanine scans of ubiquitin and histidine-tagged ubiquitin. All residues of ubiquitin other than alanine and glycine were replaced by alanine one by one, and cells expressing the indicated ubiquitin derivative in place of endogenous ubiquitin were tested for viability by growth on plates containing FOA and for the *gal* ^−^ phenotype by growth on galactose plates containing the respiration inhibitor Antimycin A. − indicates lack of growth and + indicates growth.
(TIF)

Figure S3 The over-expression of Gal3 dosage compensates the *gal* ^−^ phenotype of all ubiquitin mutants. Ten-fold serial dilutions of *SUB288GAL3ΔWL* cells expressing the indicated ubiquitin derivative in place of endogenous ubiquitin that contained *RS315* (odd lanes) or that over-expressed Gal3 from *RS315* under the control of the *ACT1* promoter (even lanes) were 10-fold serially diluted, titrated onto the indicated plates, and incubated for 6 d at 28°C. The Galactose+AA plate contained 1 mg/l Antimycin A.
(TIF)

Figure S4 The additional gene deletion of *GAL80* suppresses the *gal^−^* phenotype of all *gal^−^* ubiquitin mutants. Ten-fold serial dilutions of SUB288GAL3ΔWL cells expressing the indicated ubiquitin derivative in place of endogenous ubiquitin that contained the GAL80 gene (odd lanes) or that lacked the GAL80 gene (even lanes) were 10-fold serially diluted, titrated onto the indicated plates, and incubated for 3 d at 28°C. The Galactose+AA plate contained 1 mg/l Antimycin A.
(TIF)

Figure S5 HA-Gal80 is stable in the *gal* ^−^ *H_10_Ub* mutant strains. Left panels: *SUB288GAL3ΔL* cells expressing the indicated H_10_Ub derivatives in place of endogenous ubiquitin were transformed with the single-copy vector *RS316* expressing HA-Gal80 under the control of the *ACT1* promoter. Cells were grown in glucose liquid media to OD_600 nm_ = 1 and induced with galactose liquid media for 1 h. Cycloheximide was added at time = 0 and the amount of Gal80 protein remaining in the cells after the indicated number of hours was determined by Western blot with the help of an anti-HA antibody (upper panels). The membranes were stripped and reprobed with an anti-CPY antibody (middle panels), followed by a second stripping and staining with Coomassie Blue as loading controls (lower panels). Right panels: The ratio of the amount of HA-Gal80 protein to the loading controls for each time point was determined with Image J. The ratio of the band intensities before the addition of cycloheximide (time = 0) was set as 1 and the error bars indicate the deviations between duplicates.
(TIF)

Figure S6 GST-Gal80, but not GST, co-precipitates Skp1-HA_3_-H_10_. Endogenous Skp1 of *BY4741ΔW* cells was tagged with three HA epitopes and 10 histidines. GST (lanes 1, 2, 5, 6) and GST-Gal80 (lanes 3, 4, 7, 8) were expressed in these cells under the control of the *ACT1* promoter. Cells were grown with glucose liquid media to OD_600 nm_ = 1 (odd lanes) and induced in galactose liquid media for 1 h (even lanes). GST and GST-Gal80 were pulled down from cell extracts with the help of glutathione beads, and Inputs (lanes 1 to 4) and GST Pulldowns (lanes 5 to 8) were analyzed by Western blots with the help of an anti-HA antibody (upper panel) and an anti-GST-antibody (middle panel). The membrane was stripped and stained with Coomassie in order to compare the amount of protein loaded for Input and GST Pulldown (bottom panel).
(TIF)

Figure S7 Das1 and Ufo1 target Gal80. (A) HA-tagged Gal80 was expressed in *BY4741ΔW* (lanes 1 to 8), *BY4741ΔWΔDAS1* (lanes 9 to 16), and *BY4741ΔWΔUFO1* (lanes 17 to 24) cells from the single-copy vector *RS316* under the control of the *ACT1* promoter. Cells were grown in glucose liquid media to OD_600 nm_ = 1 and induced with galactose liquid media for 1 h. Cycloheximide was added at time = 0 and the amount of Gal80 protein remaining in the cells after the indicated number of hours was determined by Western blot with the help of an anti-HA antibody (upper panels). The membranes were stripped and stained with Coomassie Blue as loading controls (lower panels). (B) The ratio of the amount of HA-Gal80 protein to total protein (Coomassie) in *BY4741ΔW* cells (white bars), *BY4741ΔWΔDAS1* cells (black bars), and *BY4741ΔWΔUFO1* cells (grey bars) for each time point in part A was determined with Image J. The ratio of the band intensities before the addition of cycloheximide (time = 0) was set as 1 and the error bars indicate the deviations between replicates. (C) *BY4741ΔW* cells of the indicated genotype were 10-fold serially diluted, titrated onto the indicated plates, and incubated at 28°C for 3 d on the glucose plate and for 6 d on the galactose+AA ( = 0.1 mg/l Antimycin A) plate. (D) HA3-tagged Das1 and GST (lanes 1, 2, 6, 7) or GST-Gal80 (lanes 3, 4, 8, 9) were expressed in *BY4741ΔW* cells under the control of the *ACT1* promoter from the multi-copy vectors *RS423* and *RS424*, respectively. Cells were grown with glucose liquid media to OD_600 nm_ = 1 (odd lanes) and induced in galactose liquid media for 1 h (even lanes). GST and GST-Gal80 were pulled down from cell extracts with the help of glutathione beads, and Inputs and GST Pulldowns were analyzed by Western blots with the help of an anti-HA antibody (upper panel) and an anti-GST-antibody (lower panel). The size marker (M) was loaded into lane 5 and the dots indicate the positions of the marker bands of 150 kD, 100 kD, 75 kD (upper panel) and 100 kD, 75 kD, 50 kD, 37 kD (two dots), and 25 kD (lower panel). (E) HA3-tagged Ufo1 and GST (lanes 1, 2, 6, 7) or GST-Gal80 (lanes 3, 4, 8, 9) were expressed in *BY4741ΔW* cells under the control of the *ACT1* promoter from the multi-copy vectors *RS423* and *RS424*, respectively. Cells were grown with glucose liquid media to OD_600 nm_ = 1 (odd lanes) and induced in galactose liquid media for 1 h (even lanes). GST and GST-Gal80 were pulled down from cell extracts with the help of glutathione beads, and Inputs and GST Pulldowns were analyzed by Western blots with the help of an anti-HA antibody (upper panel) and an anti-GST-antibody (lower panel). The size marker (M) was loaded into lane 5. See part D for the marker sizes. (F) *BY4741ΔW* cells of the indicated genotype were 10-fold serially diluted, titrated onto the indicated plates, and incubated at 28°C for 6 d. Cells in line 3 over-expressed Ufo1 and cells in line 4 over-expressed Mdm30 from the *ACT1* promoter. The galactose plate contained 1 mg/l Antimycin A (AA).
(TIF)

Figure S8 hSrb7 interacts with hSkp1 in vitro and in vivo. (A) The Split-Ubiquitin assay. Two proteins of interest X and Y are fused to the N-terminal half of ubiquitin and to the C-terminal half of ubiquitin extended by the RUra3 reporter, Nub and Cub-RUra3, respectively. The protein interaction between X and Y brings the two halves of ubiquitin into close proximity, which causes Ubiquitin-specific proteases (Ubps) to cleave off RUra3, which is subsequently degraded by the enzymes of the N-end rule. The protein interaction between X and Y can therefore be selected for on plates containing 5-fluoro orotic acid (FOA), as Ura3 (orotidine-5′-phosphate decarboxylase) converts FOA into toxic fluoro uracil. (B) *JD52* cells expressing the indicated fusions were 10-fold serial diluted, titrated onto the depicted plates, and incubated for 3 d. Protein interaction is revealed by growth on the FOA plate and lack of growth on the uracil-depleted plate. (C) GST fusions purified from *E. coli* extracts with glutathione beads were incubated with *E. coli* extract containing H6-HA-hSrb7 (lanes 2, 3) and with *S. cerevisiae* extract containing Nub-HA-hSkp1 (lanes 5, 6). Precipitates were washed five times and analyzed by Western blot with an anti-HA antibody. (D) The human Split-Ubiquitin system. Two proteins of interest X and Y are fused to the N-terminal half of ubiquitin and to the C-terminal half of ubiquitin extended by the RGpt2 reporter, Nub and Cub-RGpt2, respectively. The protein interaction between X and Y brings the two halves of ubiquitin into close proximity, which causes Ubiquitin-specific proteases (Ubps) to cleave off RGpt2, which is subsequently degraded by the enzymes of the N-end rule. The protein interaction between X and Y causes sensitivity to hypoxanthine/aminopterin/thymine (HAT) media and resistance to 6-thioguanine (6TG). (E) Human HT1080HPRT^−^ fibroblast cells stably expressing hSrb7-Cub-RGpt2 and Nub (left panels) or hSrb7-Cub-RGpt2 and Nub-hSkp1 (right panels) were placed into control media (top panels), into HAT media (middle panels), or into media containing 6TG (bottom panels). In HAT media, the cells require functional Gpt2 (hypoxanthine-guanine phosphoribosyltransferase) enzyme, whereas 6TG media counter-selects against Gpt2 function. Growth in 6TG media and lack of growth in HAT media indicates that hSrb7 interacts with hSkp1 inside the tissue culture cells.
(TIF)

Figure S9 Mediator interacts with SCF E3 ubiquitin ligases. (A) Whole cell extracts of HeLa cells expressing myc-tagged hSkp1 (lanes 1 to 3) were incubated with anti-myc beads (lane 3). Whole cell extracts from untransfected HeLa cells (lanes 4 to 10) were incubated with an anti-hCul1 antibody (lanes 5 and 8) and with an anti-hMed6 antibody (lane 10). Antibodies were precipitated with Protein G-coupled beads. Inputs and precipitates were analyzed by Western blot with an anti-hMed6 antibody (lanes 1 to 5) and with an anti-hCul1 antibody (lanes 6 to 10). (B) HeLa cells were transfected with empty vector (Control) or with pSuper containing siRNA against *hSRB7* and *hSKP1*. Real-time PCR was used to quantify the amount of *hSRB7* mRNA and *hSKP1* mRNA relative to *GAPDH* mRNA. The value obtained for cells transformed with the empty vector was set as 1 and the error bars indicate the standard deviations between three replicates. (C) HeLa cells transfected with pSuper (control) or with pSuper containing siRNA against hSRB7 and hSKP1 were grown at 37°C (No Heat-Shock) or placed at 42°C for 15 min 1 h prior to RNA isolation (Heat-Shock). Real-time PCR was used to quantify the amount of *HSP70B* mRNA relative to *GAPDH* mRNA. The value obtained for non-heat-shocked cells transformed with pSuper was set as 1 and the error bars indicate the standard deviations between three replicates.
(TIF)

Figure S10 Snf1 does not interact with Gal80. (A) HA-Gal80 is functional. *BY4742ΔW* cells of the indicated genotype were 10-fold serially diluted, dropped onto the depicted plates, and incubated at 28°C for 6 d. The cells in the top three lines contained *RS317* and the cells in the bottom line expressed HA-Gal80 from *RS317* under the control of the *ACT1* promoter. The Galactose+AA plate contained 0.1 mg/l Antimycin A. (B) Snf1-HA_3_-H_10_ is functional. *BY4742ΔW* and *BY4742ΔWΔSNF1* cells transformed with the *LEU2*-marked single-copy vector *YCplac111* as well as *BY4742ΔW* cells expressing Snf1 chromosomally tagged with three HA epitopes and 10 histidines were 10-fold serial diluted and titrated onto the indicated plates and incubated at 28°C for 6 d. (C) *BY4742ΔW* cells transformed with *YCplac111* (lanes 1, 5) and *BY4742ΔW* cells expressing Snf1-HA_3_-H_10_ from the endogenous *SNF1* locus (lanes 2, 3, 4, 6, 7, 8) were transformed with *RS317* (lanes 1, 2, 5, 6) or with *RS317* expressing HA-Gal80 from the *ACT1* promoter (lanes 3, 4, 7, 8). Cells were grown in glucose liquid media to OD_600 nm_ = 1 (lanes 1, 2, 3, 6, 7) and induced in galactose liquid media for 1 h (lanes 4, 8). Snf1-HA_3_-H_10_ was pulled down from cell extracts with Ni-beads and Inputs (lanes 1 to 4) and Ni-pulldowns (lanes 5 to 8) were analyzed by Western blot with the help of an HA antibody (upper panel). The membrane was stripped and stained with Coomassie as a loading control (lower panel).
(TIF)

Figure S11 Snf1 interacts with Med6, Med10, Srb6, and Srb11. *JD52* cells expressing Snf1-Cub-RUra3 from the *SNF1* locus in place of endogenous Snf1 were transformed with the indicated Nub fusions. Ten-fold serial dilutions of cells were titrated onto the depicted plates and incubated at 28°C for 3 d (Glucose, Glucose−Uracil, Galactose−Uracil) or 6 d (Glucose+FOA). Protein-protein interaction between Snf1 and the Mediator subunits is indicated by growth on the FOA plate and lack of growth on the uracil-depleted plate. Functionality of the Snf1-Cub-RUra3 fusion is indicated by the ability of the strain to utilize galactose.
(TIF)

Figure S12 The Mediator subunits Srb10 and Srb11 are required for the galactose-induced protein degradation of Gal80. (A) *BY4742ΔSRB10* (lanes 1 to 12) and *BY4742ΔSRB11* (lanes 13 to 24) cells expressing HA-tagged Gal80 from *RS317* under the control of the *ACT1* promoter were grown in glucose liquid media to OD_600 nm_ = 1 (lanes 1 to 6 and 13 to 18) and induced with galactose liquid media for 1 h (lanes 7 to 12 and 19 to 24). Cycloheximide was added at time = 0 and the amount of Gal80 protein remaining in the cells after the indicated number of minutes was determined by Western blot with the help of an anti-HA antibody (upper panels). The membranes were stripped and reprobed with an anti-CPY antibody (middle panels), followed by a second stripping and staining with Coomassie Blue as loading controls (lower panels). (B) The ratio of the amount of HA-Gal80 protein to CPY protein in *BY4742ΔW* cells (white bars), *BY4742ΔSRB10* cells (black bars), and *BY4742ΔSRB11* cells (grey bars) for each time point in part A was determined with Image J. The ratio of the band intensities before the addition of cycloheximide (time = 0) was set as 1 and the error bars indicate the deviations between duplicates. The Western blots for the *BY4742ΔW* wild-type control are presented in Figures 4C (lanes 1–12) and 6A (lanes 1–12). (C) Ten-fold serial dilutions of the indicated strains were titrated onto the depicted plates and incubated at 28°C for 3 d. The galactose plates contained 1 mg/l of the respiration inhibitor Antimycin A.
(TIF)

Figure S13 Galactose induction of *GAL1* mRNA is restored in the *ΔMDM30* strain, if the cells are pre-grown with raffinose instead of with glucose. (A) *BY4741ΔW* wild-type and *ΔMDM30* cells were grown in glucose liquid media to OD_600 nm_ = 1 (Glu) and induced with galactose liquid media for 8 h (Gal 8 h). Total RNA was isolated and *GAL1* mRNA was determined relative to *ACT1* mRNA by quantitative real-time PCR. The value determined for *BY4741ΔW* wild-type cells grown with glucose liquid media was set as 1 and the error bars indicate the standard deviations between three replicates. (B) *BY4741ΔW* wild-type and *ΔMDM30* cells were grown in raffinose liquid media to OD_600 nm_ = 1 (Raf) and induced with galactose liquid media for 1 h (Gal 1 h). Total RNA was isolated and *GAL1* mRNA was determined relative to *ACT1* mRNA by quantitative real-time PCR. The value determined for *BY4741ΔW* wild-type cells grown with raffinose liquid media was set as 1 and the error bars indicate the standard deviations between three replicates.
(TIF)

Figure S14 Protein degradation of Gal80 is restored in the *ΔMDM30* strain, if the cells are pre-grown with raffinose instead of with glucose. (A) HA-tagged Gal80 was expressed in *BY4741ΔW* and *BY4741ΔWΔMDM30* cells from the single-copy vector *RS316* under the control of the *ACT1* promoter. Cells were grown in raffinose liquid media (lanes 1 to 4 and 9 to 12) to OD_600 nm_ = 1 and induced with galactose liquid media for 1 h (lanes 5 to 8 and 13 to 16). Cycloheximide was added at time = 0 and the amount of Gal80 protein remaining in the cells after the indicated number of hours was determined by Western blot with the help of an anti-HA antibody (upper panels). The membranes were stripped and reprobed with an anti-CPY antibody (middle panels), followed by a second stripping and staining with Coomassie Blue as loading controls (lower panels). (B) The ratio of the amount of HA-Gal80 protein to CPY protein in *BY4741ΔW* cells (white bars) and *BY4741ΔWΔMDM30* cells (black bars) for each time point in part A was determined with Image J. The ratio of the band intensities before the addition of cycloheximide (time = 0) was set as 1 and the error bars indicate the deviations between duplicates.
(TIF)

Figure S15 Gal3 is not required for the galactose-stimulated protein degradation of Gal80. (A) *BY4741ΔW* cells of the indicated genotype were 10-fold serially diluted, dropped onto the depicted plates, and incubated at 28°C for 3 d. The titrations were performed in duplicates. The Galactose+AA plate contained 1 mg/l Antimycin A. (B) HA-tagged Gal80 was expressed in *BY4741ΔWΔGAL3* cells from the single-copy vector *RS316* under the control of the *ACT1* promoter. Cells were grown in glucose liquid media (lanes 1 to 4) to OD_600 nm_ = 1 and induced with galactose liquid media for 1 h (lanes 5 to 8). Cycloheximide was added at time = 0 and the amount of Gal80 protein remaining in the cells after the indicated number of hours was determined by Western blot with the help of an anti-HA antibody (upper panel). The membranes were stripped and reprobed with an anti-CPY antibody (middle panel), followed by a second stripping and staining with Coomassie Blue as loading control (lower panel). (C) The ratio of the amount of HA-Gal80 protein to CPY protein for each time point in part B was determined with Image J. The ratio of the band intensities before the addition of cycloheximide (time = 0) was set as 1 and the error bars indicate the deviations between duplicates.
(TIF)

Figure S16 The degradation of Gal80 as the limiting factor for the activation of the *GAL1* gene. (A) *BY4741ΔW* cells were grown in glucose liquid media to OD_600 nm_ = 1 (0 h) and induced with galactose liquid media for the indicated number of hours. Total RNA was isolated and *GAL1* mRNA was determined relative to *ACT1* mRNA by quantitative real-time PCR. The value determined for *BY4741ΔW* cells grown with glucose liquid media was set as 1 and the error bars indicate the standard deviations between three replicates. (B) *BY4741ΔW* cells were grown in raffinose liquid media to OD_600 nm_ = 1 (0 min) and induced with galactose liquid media for the indicated number of minutes. Total RNA was isolated and *GAL1* mRNA was determined relative to *ACT1* mRNA by quantitative real-time PCR. The value determined for *BY4741ΔW* cells grown with raffinose liquid media was set as 1 and the error bars indicate the standard deviations between three replicates.
(TIF)

Table S1 Genotypes and sources of strains used in this study.
(DOC)

Table S2 Names of plasmids constructed for this study and DNA sequences of PCR primers used to generate these plasmids.
(DOC)

## Abbreviations

6TG: 6-thioguanine
AA: Antimycin A
CPY: carboxypeptidase Y
CTD: C-terminal domain of RNA Polymerase II
Cub: C-terminal half of ubiquitin
FOA: 5-fluoro orotic acid
Gal: galactose
gal-: galactose-utilization defective
GEM: gene expression machine
Glu: glucose
Gpt2: hypoxanthine-guanine phosphoribosyltransferase
GST: glutathione-S-transferase
GTF: general transcription factor
HA: hemagglutinin
HAT: hypoxanthine/aminopterin/thymine
Nub: N-terminal half of ubiquitin
PBS: phosphate-buffered saline
PCR: polymerase chain reaction
Raf: raffinose
SCF: Skip1-Cullin-F-box protein
TBP: TATA-binding protein
Ubp: ubiquitin-specific protease
UPD: ubiquitin proteasome-dependent degradation
Ura3: orotidine-5′-phosphate decarboxylase
