# Supplementary figures and images for: Mediator Acts Upstream of the Transcriptional Activator Gal4
Source: PLoS Biol. 2012 Mar 27;10(3):e1001290. doi: 10.1371/journal.pbio.1001290 (PMC3313914; doi:10.1371/journal.pbio.1001290)

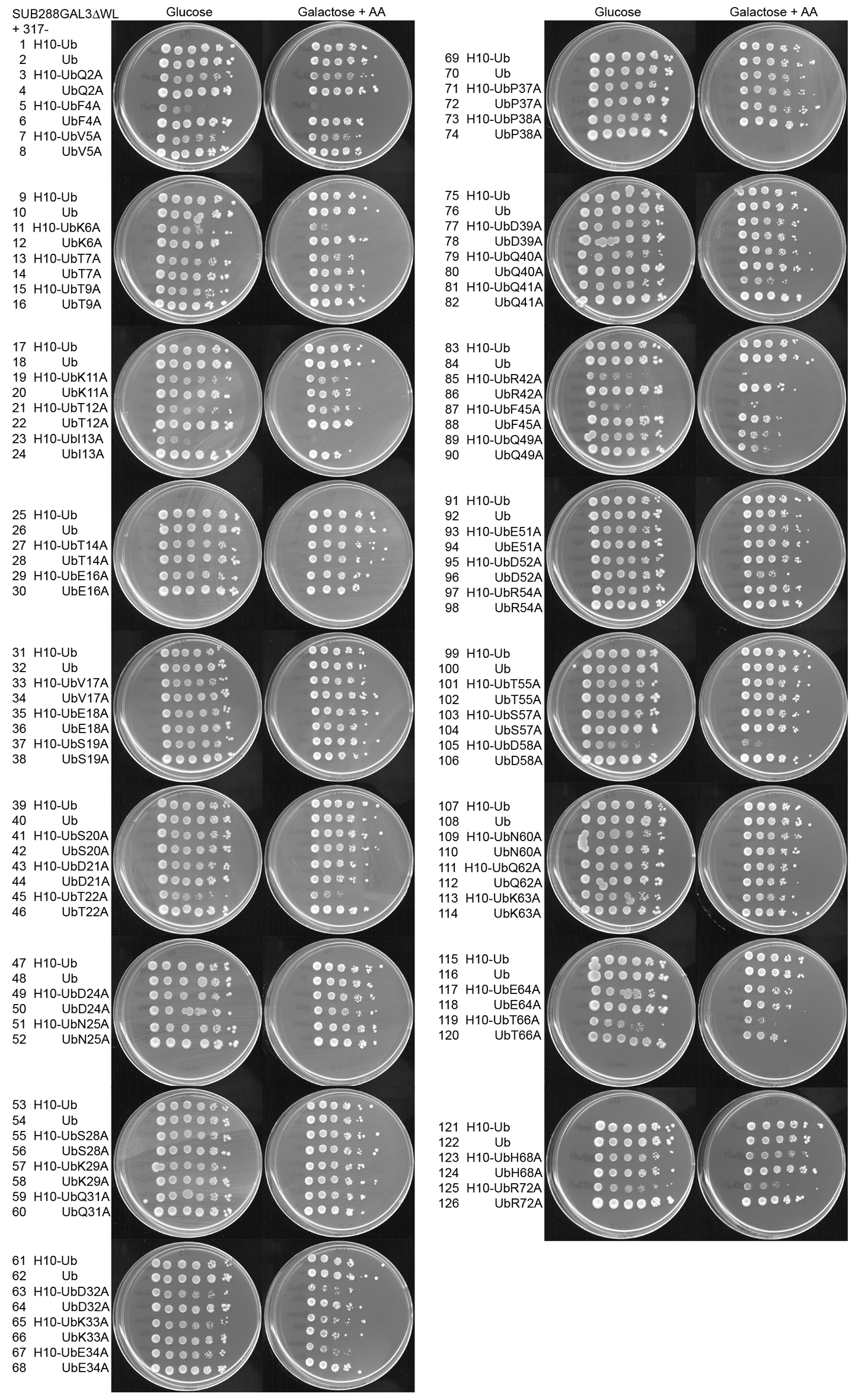

Supplement: Figure S1 — Identification of galactose utilization-defective Ub mutants. Ten-fold serial dilutions of SUB288GAL3ΔWL cells expressing the indicated ubiquitin derivatives in place of endogenous ubiquitin were titrated onto the depicted plates and incubated at 28°C for 6 d. The ubiquitin derivatives were expressed from the LYS2-marked single-copy vector RS317 under the control of the ACT1 promoter. The galactose plates contained 1 mg/l of the respiration inhibitor Antimycin A (AA). (TIF) [file pbio.1001290.s001.tif]

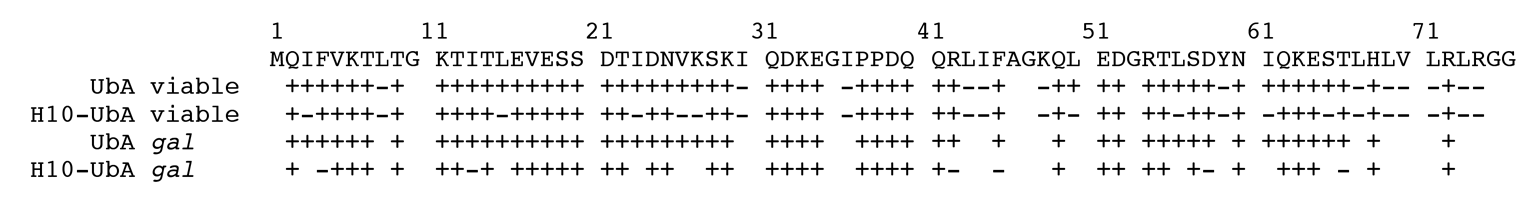

Supplement: Figure S2 — Summary of the results of the alanine scans of ubiquitin and histidine-tagged ubiquitin. All residues of ubiquitin other than alanine and glycine were replaced by alanine one by one, and cells expressing the indicated ubiquitin derivative in place of endogenous ubiquitin were tested for viability by growth on plates containing FOA and for the gal − phenotype by growth on galactose plates containing the respiration inhibitor Antimycin A. − indicates lack of growth and + indicates growth. (TIF) [file pbio.1001290.s002.tif]

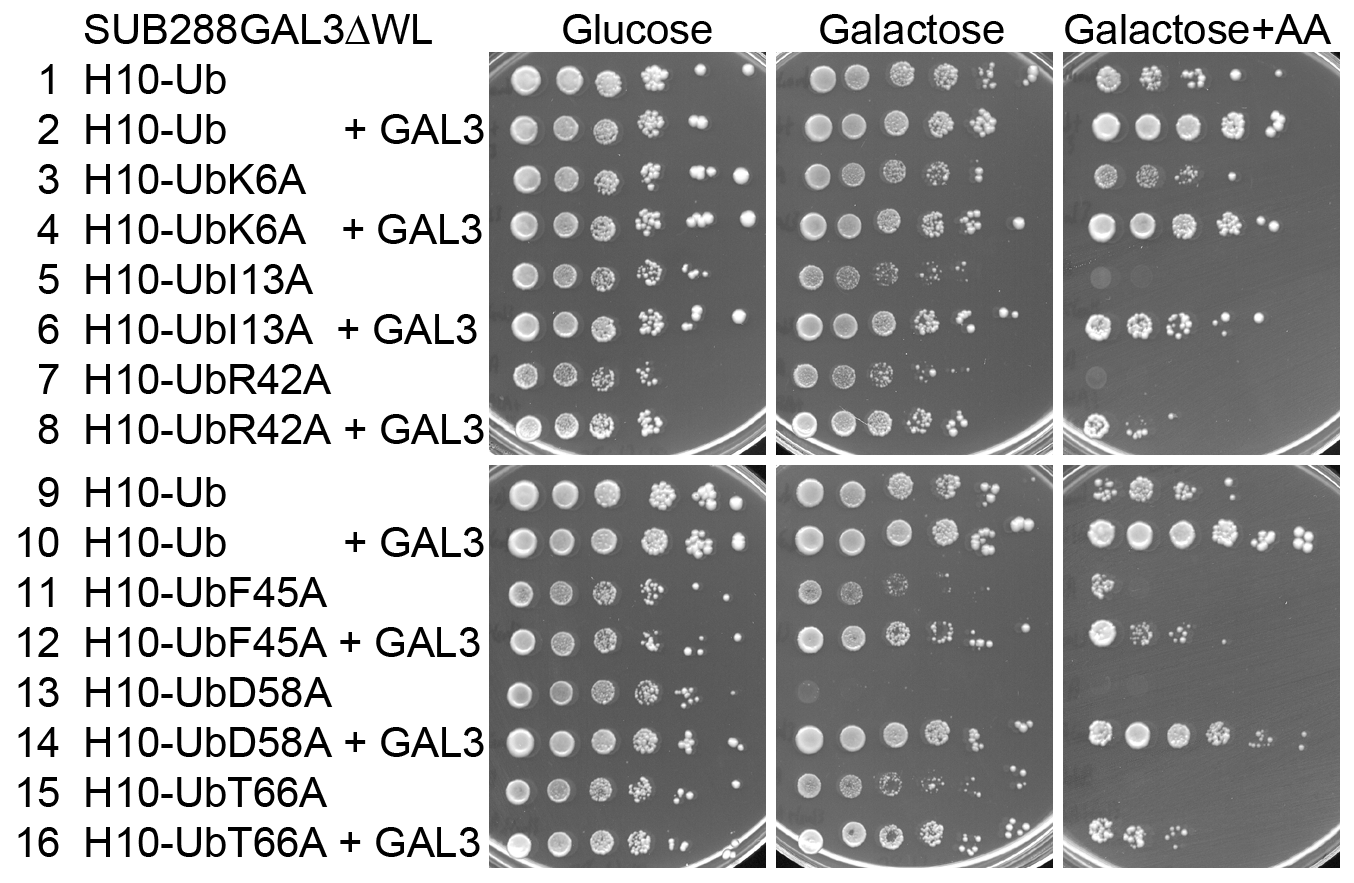

Supplement: Figure S3 — The over-expression of Gal3 dosage compensates the gal − phenotype of all ubiquitin mutants. Ten-fold serial dilutions of SUB288GAL3ΔWL cells expressing the indicated ubiquitin derivative in place of endogenous ubiquitin that contained RS315 (odd lanes) or that over-expressed Gal3 from RS315 under the control of the ACT1 promoter (even lanes) were 10-fold serially diluted, titrated onto the indicated plates, and incubated for 6 d at 28°C. The Galactose+AA plate contained 1 mg/l Antimycin A. (TIF) [file pbio.1001290.s003.tif]

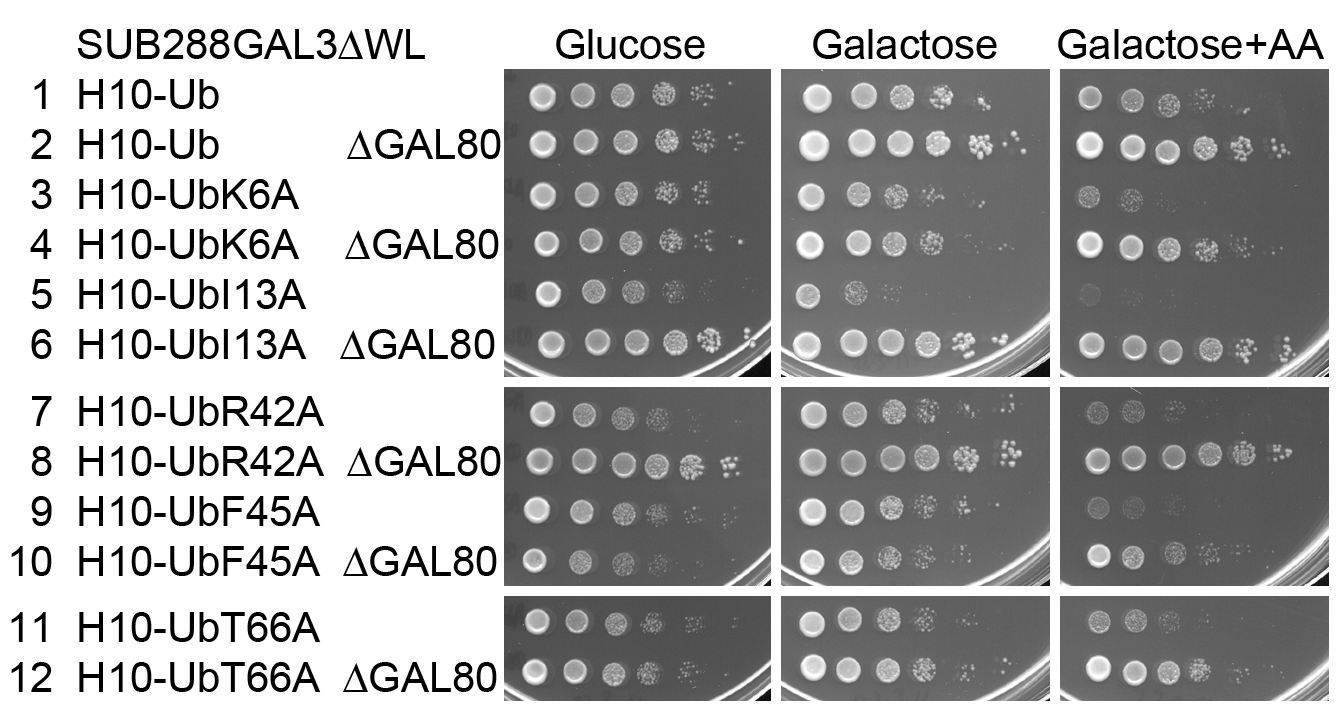

Supplement: Figure S4 — The additional gene deletion of GAL80 suppresses the gal− phenotype of all gal− ubiquitin mutants. Ten-fold serial dilutions of SUB288GAL3ΔWL cells expressing the indicated ubiquitin derivative in place of endogenous ubiquitin that contained the GAL80 gene (odd lanes) or that lacked the GAL80 gene (even lanes) were 10-fold serially diluted, titrated onto the indicated plates, and incubated for 3 d at 28°C. The Galactose+AA plate contained 1 mg/l Antimycin A. (TIF) [file pbio.1001290.s004.tif]

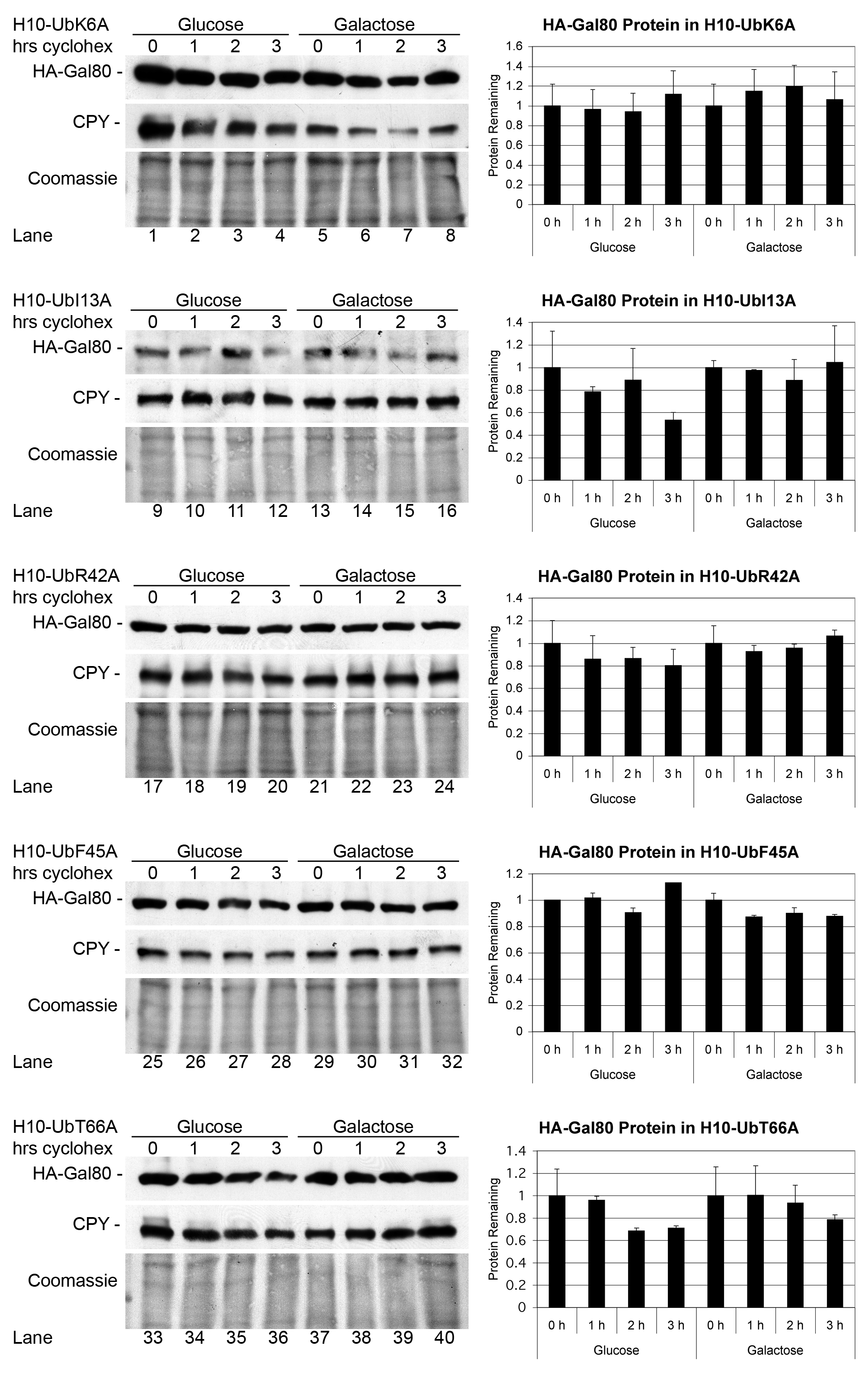

Supplement: Figure S5 — HA-Gal80 is stable in the gal − H10Ub mutant strains. Left panels: SUB288GAL3ΔL cells expressing the indicated H10Ub derivatives in place of endogenous ubiquitin were transformed with the single-copy vector RS316 expressing HA-Gal80 under the control of the ACT1 promoter. Cells were grown in glucose liquid media to OD600 nm = 1 and induced with galactose liquid media for 1 h. Cycloheximide was added at time = 0 and the amount of Gal80 protein remaining in the cells after the indicated number of hours was determined by Western blot with the help of an anti-HA antibody (upper panels). The membranes were stripped and reprobed with an anti-CPY antibody (middle panels), followed by a second stripping and staining with Coomassie Blue as loading controls (lower panels). Right panels: The ratio of the amount of HA-Gal80 protein to the loading controls for each time point was determined with Image J. The ratio of the band intensities before the addition of cycloheximide (time = 0) was set as 1 and the error bars indicate the deviations between duplicates. (TIF) [file pbio.1001290.s005.tif]

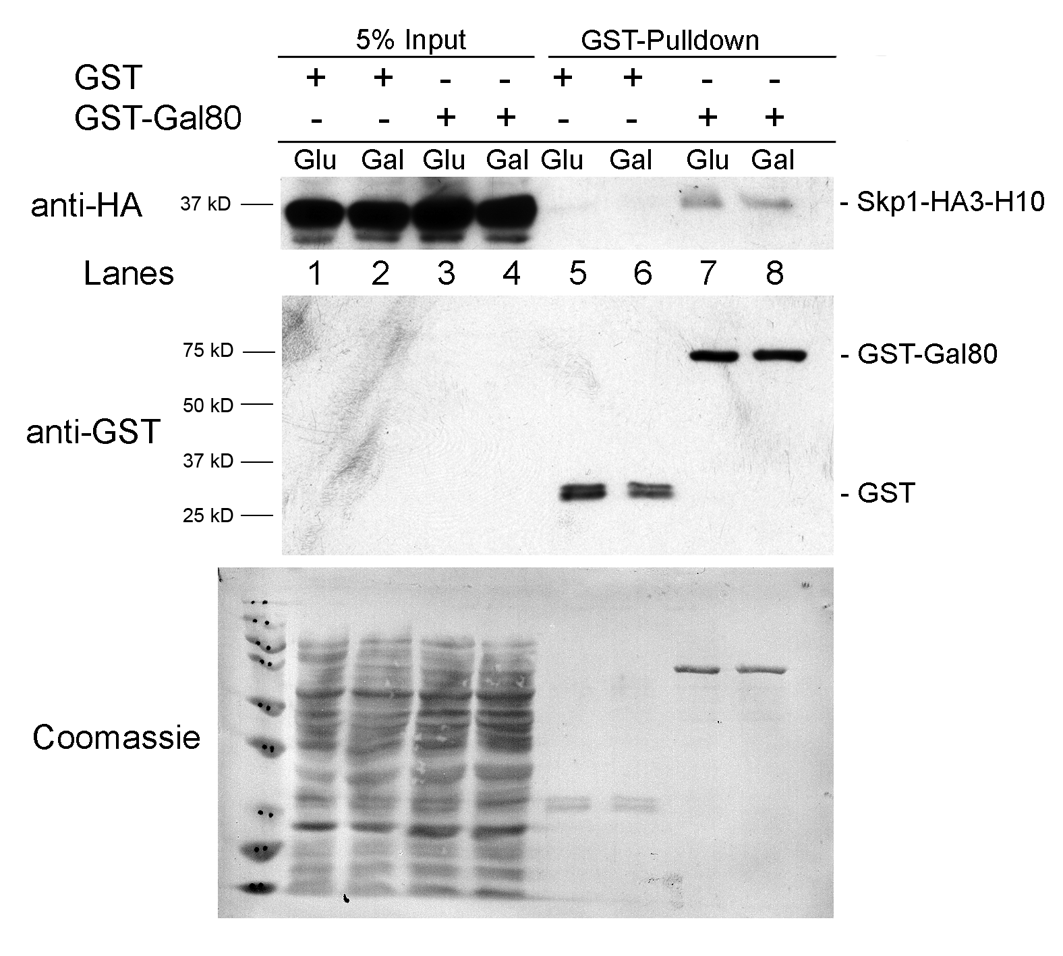

Supplement: Figure S6 — GST-Gal80, but not GST, co-precipitates Skp1-HA3-H10. Endogenous Skp1 of BY4741ΔW cells was tagged with three HA epitopes and 10 histidines. GST (lanes 1, 2, 5, 6) and GST-Gal80 (lanes 3, 4, 7, 8) were expressed in these cells under the control of the ACT1 promoter. Cells were grown with glucose liquid media to OD600 nm = 1 (odd lanes) and induced in galactose liquid media for 1 h (even lanes). GST and GST-Gal80 were pulled down from cell extracts with the help of glutathione beads, and Inputs (lanes 1 to 4) and GST Pulldowns (lanes 5 to 8) were analyzed by Western blots with the help of an anti-HA antibody (upper panel) and an anti-GST-antibody (middle panel). The membrane was stripped and stained with Coomassie in order to compare the amount of protein loaded for Input and GST Pulldown (bottom panel). (TIF) [file pbio.1001290.s006.tif]

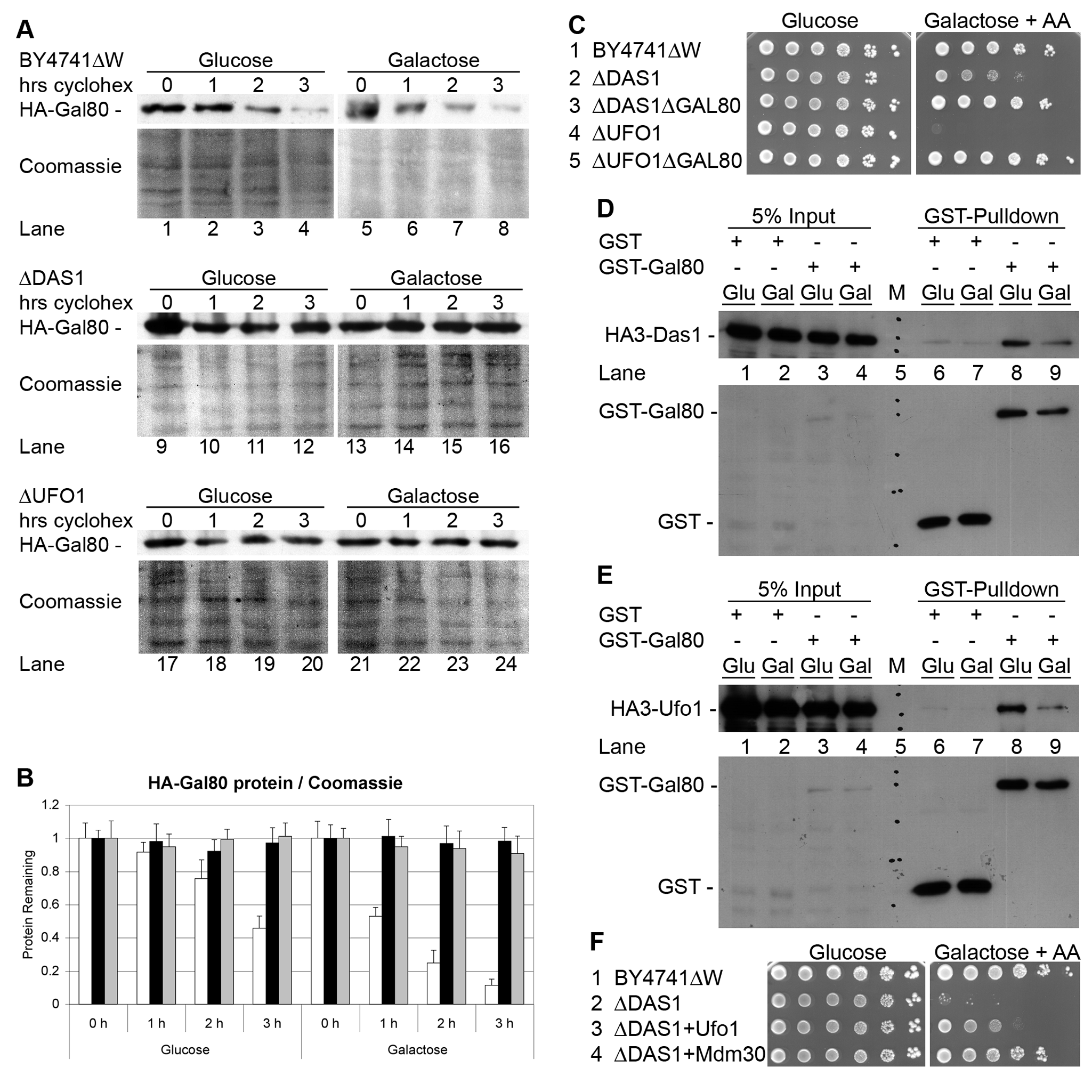

Supplement: Figure S7 — Das1 and Ufo1 target Gal80. (A) HA-tagged Gal80 was expressed in BY4741ΔW (lanes 1 to 8), BY4741ΔWΔDAS1 (lanes 9 to 16), and BY4741ΔWΔUFO1 (lanes 17 to 24) cells from the single-copy vector RS316 under the control of the ACT1 promoter. Cells were grown in glucose liquid media to OD600 nm = 1 and induced with galactose liquid media for 1 h. Cycloheximide was added at time = 0 and the amount of Gal80 protein remaining in the cells after the indicated number of hours was determined by Western blot with the help of an anti-HA antibody (upper panels). The membranes were stripped and stained with Coomassie Blue as loading controls (lower panels). (B) The ratio of the amount of HA-Gal80 protein to total protein (Coomassie) in BY4741ΔW cells (white bars), BY4741ΔWΔDAS1 cells (black bars), and BY4741ΔWΔUFO1 cells (grey bars) for each time point in part A was determined with Image J. The ratio of the band intensities before the addition of cycloheximide (time = 0) was set as 1 and the error bars indicate the deviations between replicates. (C) BY4741ΔW cells of the indicated genotype were 10-fold serially diluted, titrated onto the indicated plates, and incubated at 28°C for 3 d on the glucose plate and for 6 d on the galactose+AA ( = 0.1 mg/l Antimycin A) plate. (D) HA3-tagged Das1 and GST (lanes 1, 2, 6, 7) or GST-Gal80 (lanes 3, 4, 8, 9) were expressed in BY4741ΔW cells under the control of the ACT1 promoter from the multi-copy vectors RS423 and RS424, respectively. Cells were grown with glucose liquid media to OD600 nm = 1 (odd lanes) and induced in galactose liquid media for 1 h (even lanes). GST and GST-Gal80 were pulled down from cell extracts with the help of glutathione beads, and Inputs and GST Pulldowns were analyzed by Western blots with the help of an anti-HA antibody (upper panel) and an anti-GST-antibody (lower panel). The size marker (M) was loaded into lane 5 and the dots indicate the positions of the marker bands of 150 kD, 100 kD, 75 kD (upper panel) and 100 [file pbio.1001290.s007.tif]

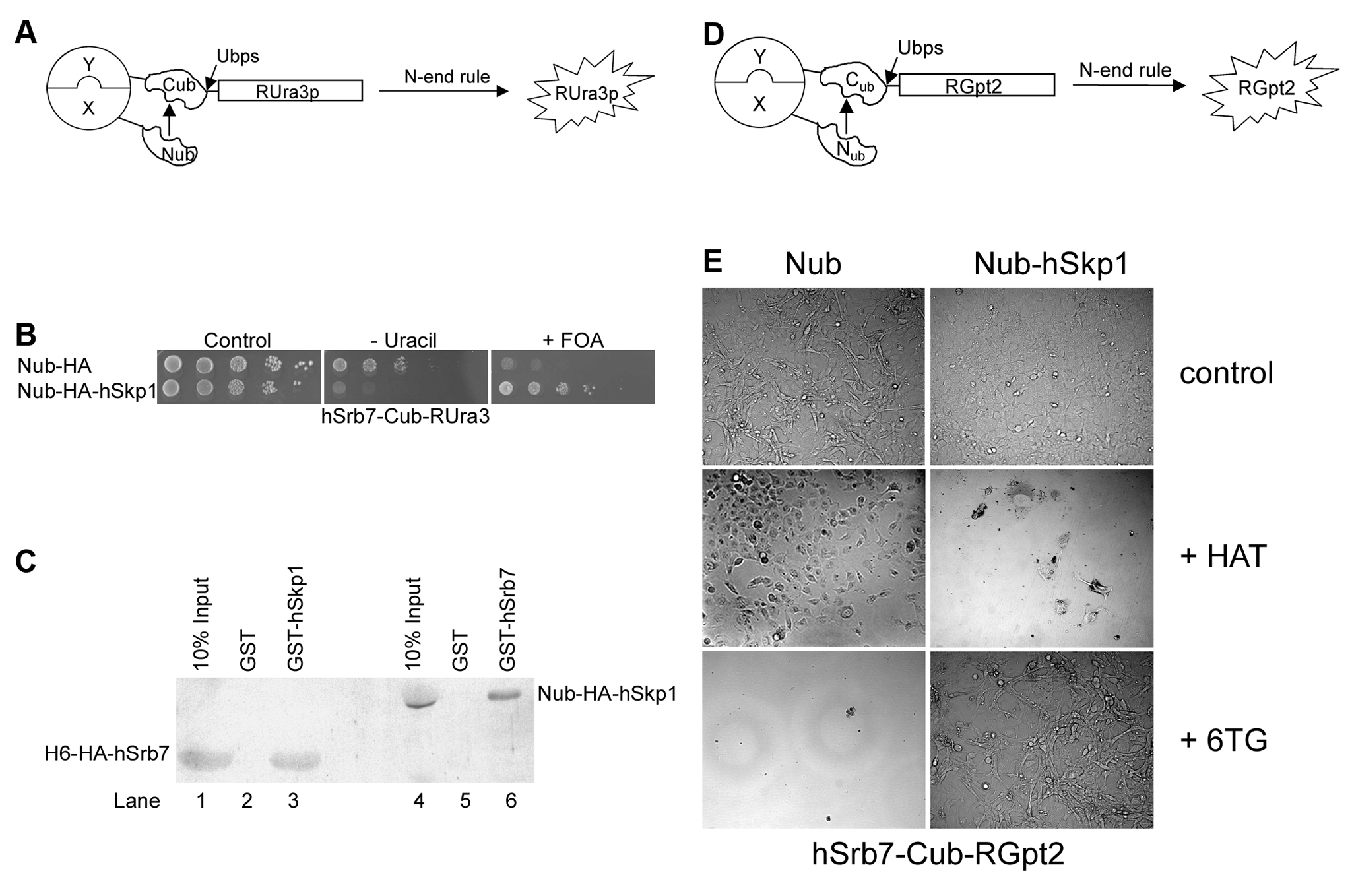

Supplement: Figure S8 — hSrb7 interacts with hSkp1 in vitro and in vivo. (A) The Split-Ubiquitin assay. Two proteins of interest X and Y are fused to the N-terminal half of ubiquitin and to the C-terminal half of ubiquitin extended by the RUra3 reporter, Nub and Cub-RUra3, respectively. The protein interaction between X and Y brings the two halves of ubiquitin into close proximity, which causes Ubiquitin-specific proteases (Ubps) to cleave off RUra3, which is subsequently degraded by the enzymes of the N-end rule. The protein interaction between X and Y can therefore be selected for on plates containing 5-fluoro orotic acid (FOA), as Ura3 (orotidine-5′-phosphate decarboxylase) converts FOA into toxic fluoro uracil. (B) JD52 cells expressing the indicated fusions were 10-fold serial diluted, titrated onto the depicted plates, and incubated for 3 d. Protein interaction is revealed by growth on the FOA plate and lack of growth on the uracil-depleted plate. (C) GST fusions purified from E. coli extracts with glutathione beads were incubated with E. coli extract containing H6-HA-hSrb7 (lanes 2, 3) and with S. cerevisiae extract containing Nub-HA-hSkp1 (lanes 5, 6). Precipitates were washed five times and analyzed by Western blot with an anti-HA antibody. (D) The human Split-Ubiquitin system. Two proteins of interest X and Y are fused to the N-terminal half of ubiquitin and to the C-terminal half of ubiquitin extended by the RGpt2 reporter, Nub and Cub-RGpt2, respectively. The protein interaction between X and Y brings the two halves of ubiquitin into close proximity, which causes Ubiquitin-specific proteases (Ubps) to cleave off RGpt2, which is subsequently degraded by the enzymes of the N-end rule. The protein interaction between X and Y causes sensitivity to hypoxanthine/aminopterin/thymine (HAT) media and resistance to 6-thioguanine (6TG). (E) Human HT1080HPRT− fibroblast cells stably expressing hSrb7-Cub-RGpt2 and Nub (left panels) or hSrb7-Cub-RGpt2 and Nub-hSkp1 (right panels) were placed [file pbio.1001290.s008.tif]

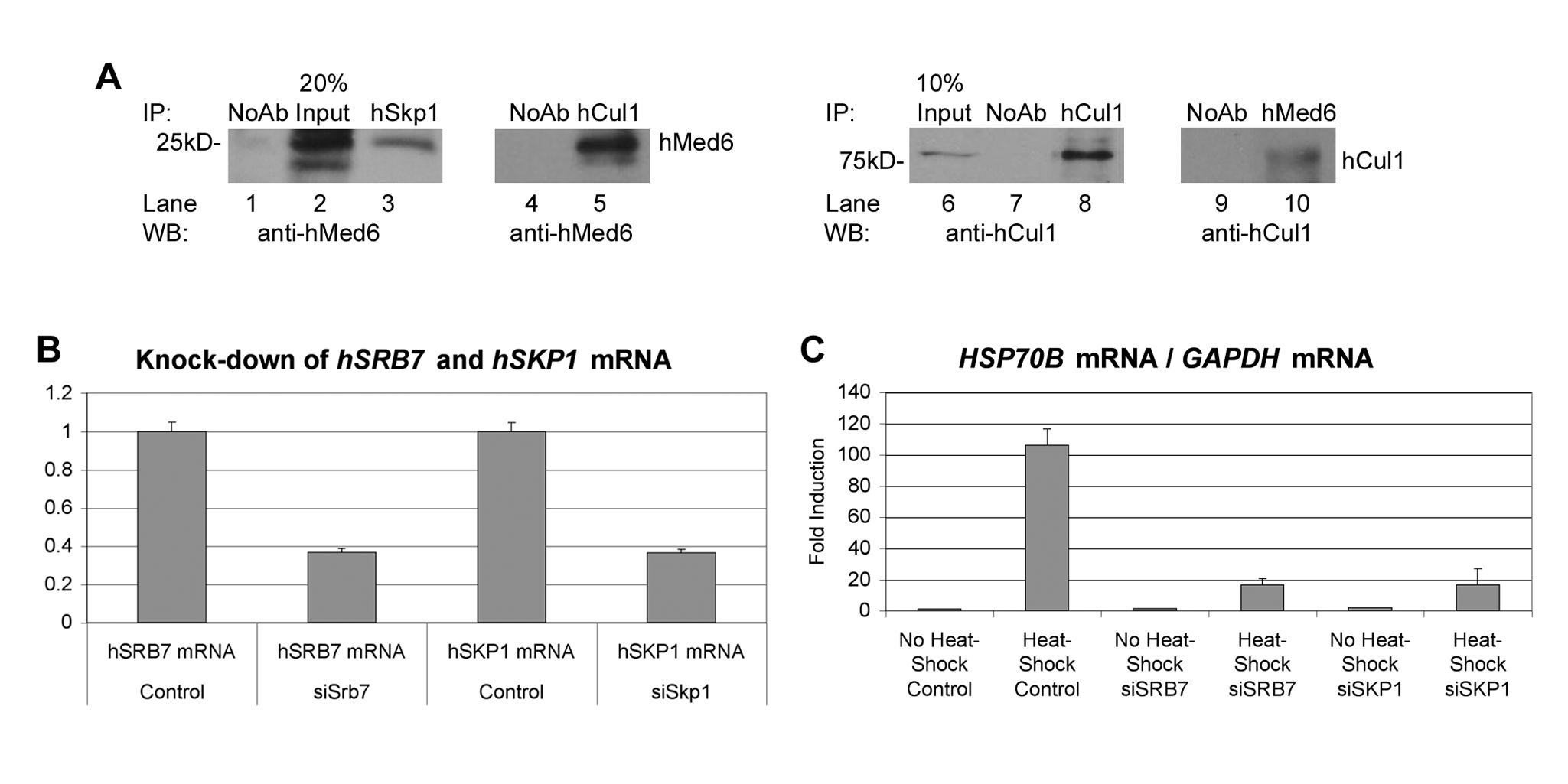

Supplement: Figure S9 — Mediator interacts with SCF E3 ubiquitin ligases. (A) Whole cell extracts of HeLa cells expressing myc-tagged hSkp1 (lanes 1 to 3) were incubated with anti-myc beads (lane 3). Whole cell extracts from untransfected HeLa cells (lanes 4 to 10) were incubated with an anti-hCul1 antibody (lanes 5 and 8) and with an anti-hMed6 antibody (lane 10). Antibodies were precipitated with Protein G-coupled beads. Inputs and precipitates were analyzed by Western blot with an anti-hMed6 antibody (lanes 1 to 5) and with an anti-hCul1 antibody (lanes 6 to 10). (B) HeLa cells were transfected with empty vector (Control) or with pSuper containing siRNA against hSRB7 and hSKP1. Real-time PCR was used to quantify the amount of hSRB7 mRNA and hSKP1 mRNA relative to GAPDH mRNA. The value obtained for cells transformed with the empty vector was set as 1 and the error bars indicate the standard deviations between three replicates. (C) HeLa cells transfected with pSuper (control) or with pSuper containing siRNA against hSRB7 and hSKP1 were grown at 37°C (No Heat-Shock) or placed at 42°C for 15 min 1 h prior to RNA isolation (Heat-Shock). Real-time PCR was used to quantify the amount of HSP70B mRNA relative to GAPDH mRNA. The value obtained for non-heat-shocked cells transformed with pSuper was set as 1 and the error bars indicate the standard deviations between three replicates. (TIF) [file pbio.1001290.s009.tif]

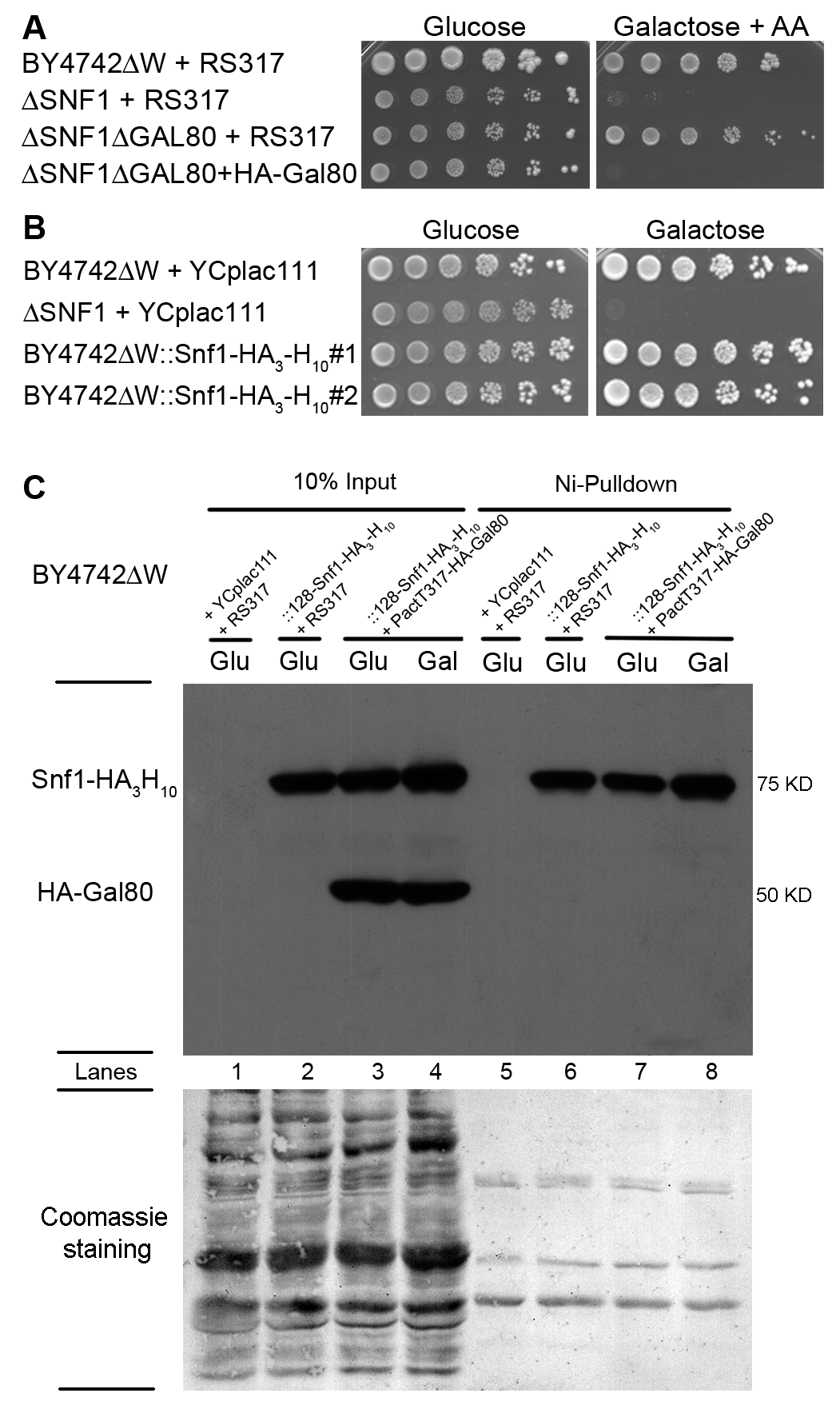

Supplement: Figure S10 — Snf1 does not interact with Gal80. (A) HA-Gal80 is functional. BY4742ΔW cells of the indicated genotype were 10-fold serially diluted, dropped onto the depicted plates, and incubated at 28°C for 6 d. The cells in the top three lines contained RS317 and the cells in the bottom line expressed HA-Gal80 from RS317 under the control of the ACT1 promoter. The Galactose+AA plate contained 0.1 mg/l Antimycin A. (B) Snf1-HA3-H10 is functional. BY4742ΔW and BY4742ΔWΔSNF1 cells transformed with the LEU2-marked single-copy vector YCplac111 as well as BY4742ΔW cells expressing Snf1 chromosomally tagged with three HA epitopes and 10 histidines were 10-fold serial diluted and titrated onto the indicated plates and incubated at 28°C for 6 d. (C) BY4742ΔW cells transformed with YCplac111 (lanes 1, 5) and BY4742ΔW cells expressing Snf1-HA3-H10 from the endogenous SNF1 locus (lanes 2, 3, 4, 6, 7, 8) were transformed with RS317 (lanes 1, 2, 5, 6) or with RS317 expressing HA-Gal80 from the ACT1 promoter (lanes 3, 4, 7, 8). Cells were grown in glucose liquid media to OD600 nm = 1 (lanes 1, 2, 3, 6, 7) and induced in galactose liquid media for 1 h (lanes 4, 8). Snf1-HA3-H10 was pulled down from cell extracts with Ni-beads and Inputs (lanes 1 to 4) and Ni-pulldowns (lanes 5 to 8) were analyzed by Western blot with the help of an HA antibody (upper panel). The membrane was stripped and stained with Coomassie as a loading control (lower panel). (TIF) [file pbio.1001290.s010.tif]

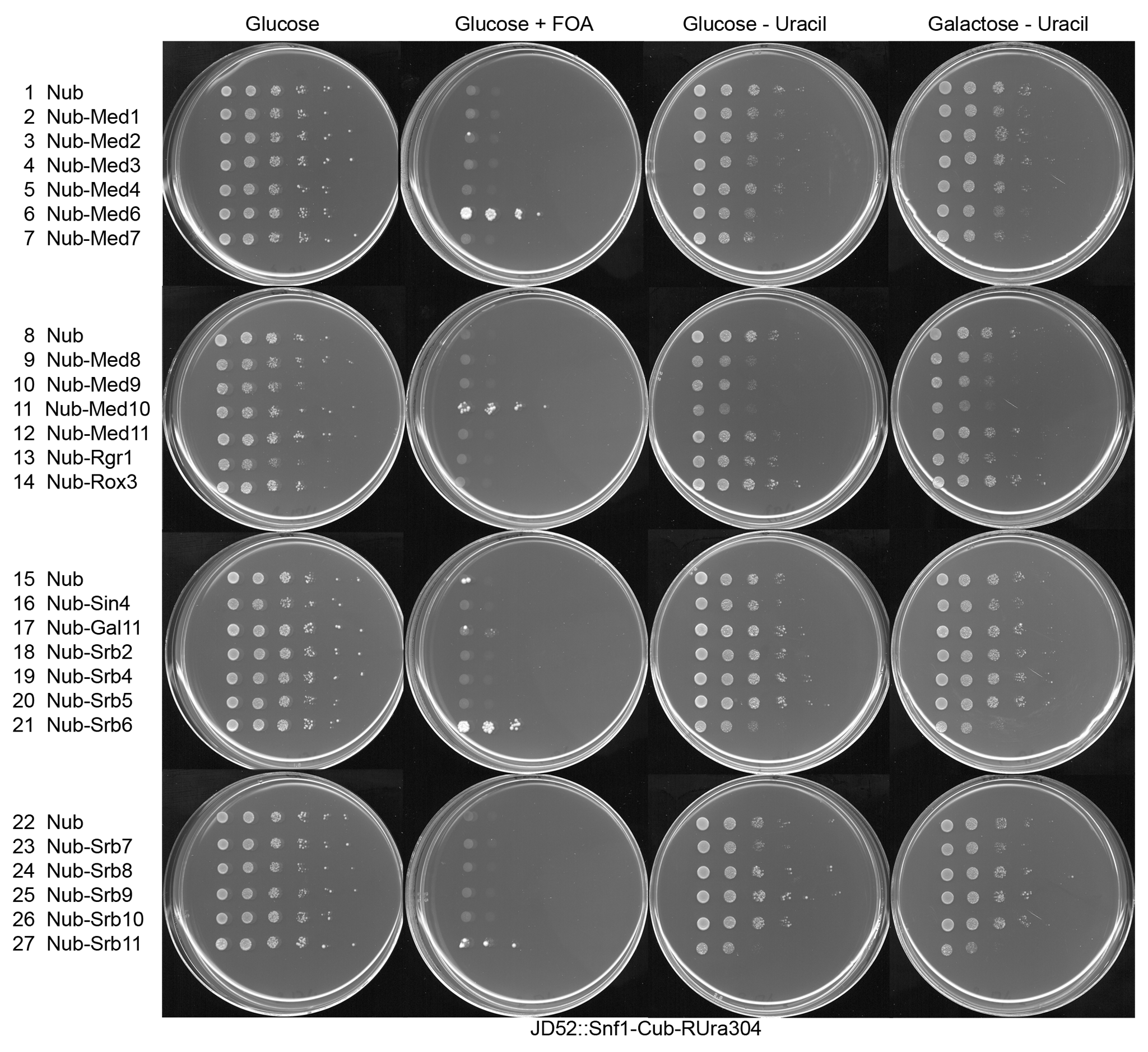

Supplement: Figure S11 — Snf1 interacts with Med6, Med10, Srb6, and Srb11. JD52 cells expressing Snf1-Cub-RUra3 from the SNF1 locus in place of endogenous Snf1 were transformed with the indicated Nub fusions. Ten-fold serial dilutions of cells were titrated onto the depicted plates and incubated at 28°C for 3 d (Glucose, Glucose−Uracil, Galactose−Uracil) or 6 d (Glucose+FOA). Protein-protein interaction between Snf1 and the Mediator subunits is indicated by growth on the FOA plate and lack of growth on the uracil-depleted plate. Functionality of the Snf1-Cub-RUra3 fusion is indicated by the ability of the strain to utilize galactose. (TIF) [file pbio.1001290.s011.tif]

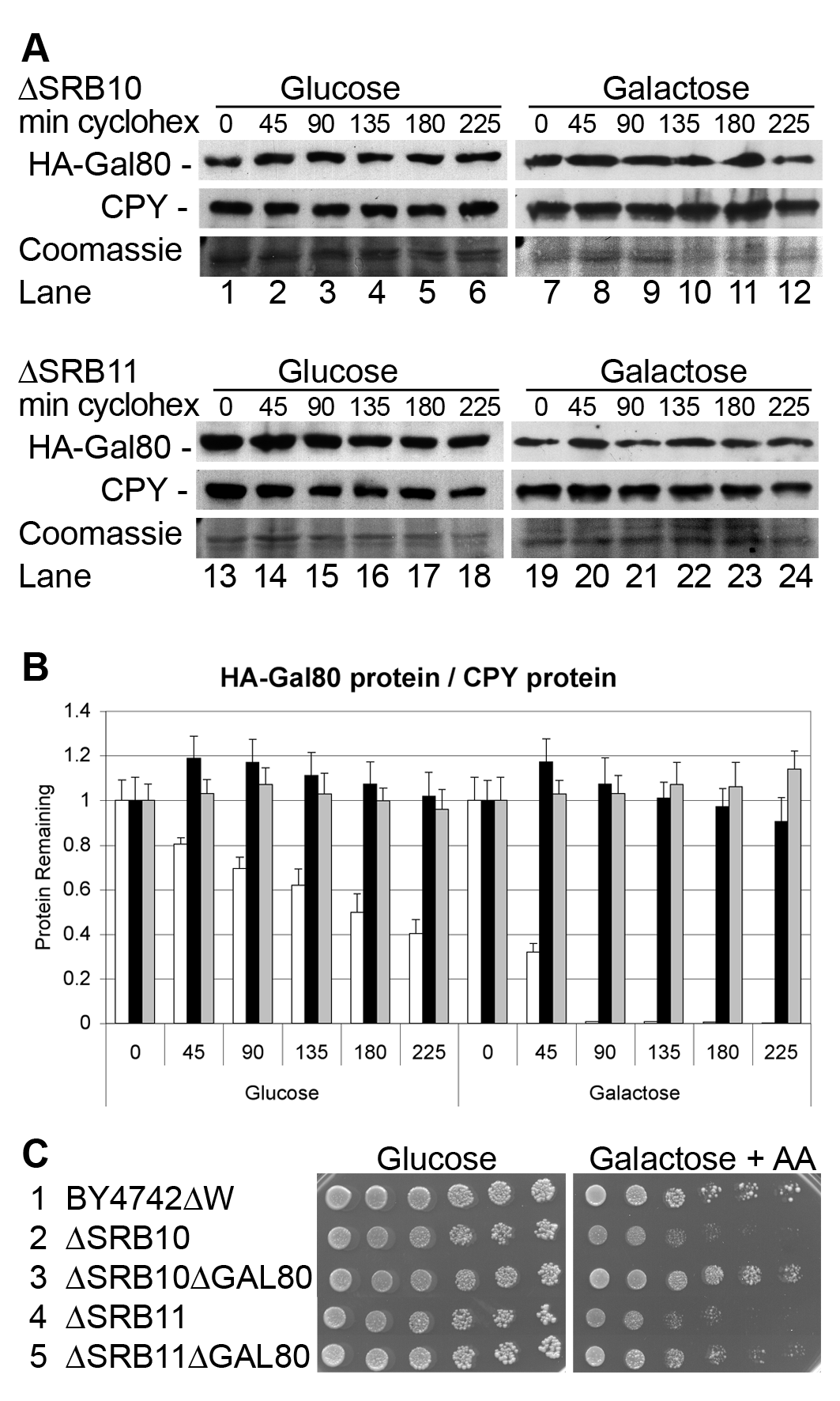

Supplement: Figure S12 — The Mediator subunits Srb10 and Srb11 are required for the galactose-induced protein degradation of Gal80. (A) BY4742ΔSRB10 (lanes 1 to 12) and BY4742ΔSRB11 (lanes 13 to 24) cells expressing HA-tagged Gal80 from RS317 under the control of the ACT1 promoter were grown in glucose liquid media to OD600 nm = 1 (lanes 1 to 6 and 13 to 18) and induced with galactose liquid media for 1 h (lanes 7 to 12 and 19 to 24). Cycloheximide was added at time = 0 and the amount of Gal80 protein remaining in the cells after the indicated number of minutes was determined by Western blot with the help of an anti-HA antibody (upper panels). The membranes were stripped and reprobed with an anti-CPY antibody (middle panels), followed by a second stripping and staining with Coomassie Blue as loading controls (lower panels). (B) The ratio of the amount of HA-Gal80 protein to CPY protein in BY4742ΔW cells (white bars), BY4742ΔSRB10 cells (black bars), and BY4742ΔSRB11 cells (grey bars) for each time point in part A was determined with Image J. The ratio of the band intensities before the addition of cycloheximide (time = 0) was set as 1 and the error bars indicate the deviations between duplicates. The Western blots for the BY4742ΔW wild-type control are presented in Figures 4C (lanes 1–12) and 6A (lanes 1–12). (C) Ten-fold serial dilutions of the indicated strains were titrated onto the depicted plates and incubated at 28°C for 3 d. The galactose plates contained 1 mg/l of the respiration inhibitor Antimycin A. (TIF) [file pbio.1001290.s012.tif]

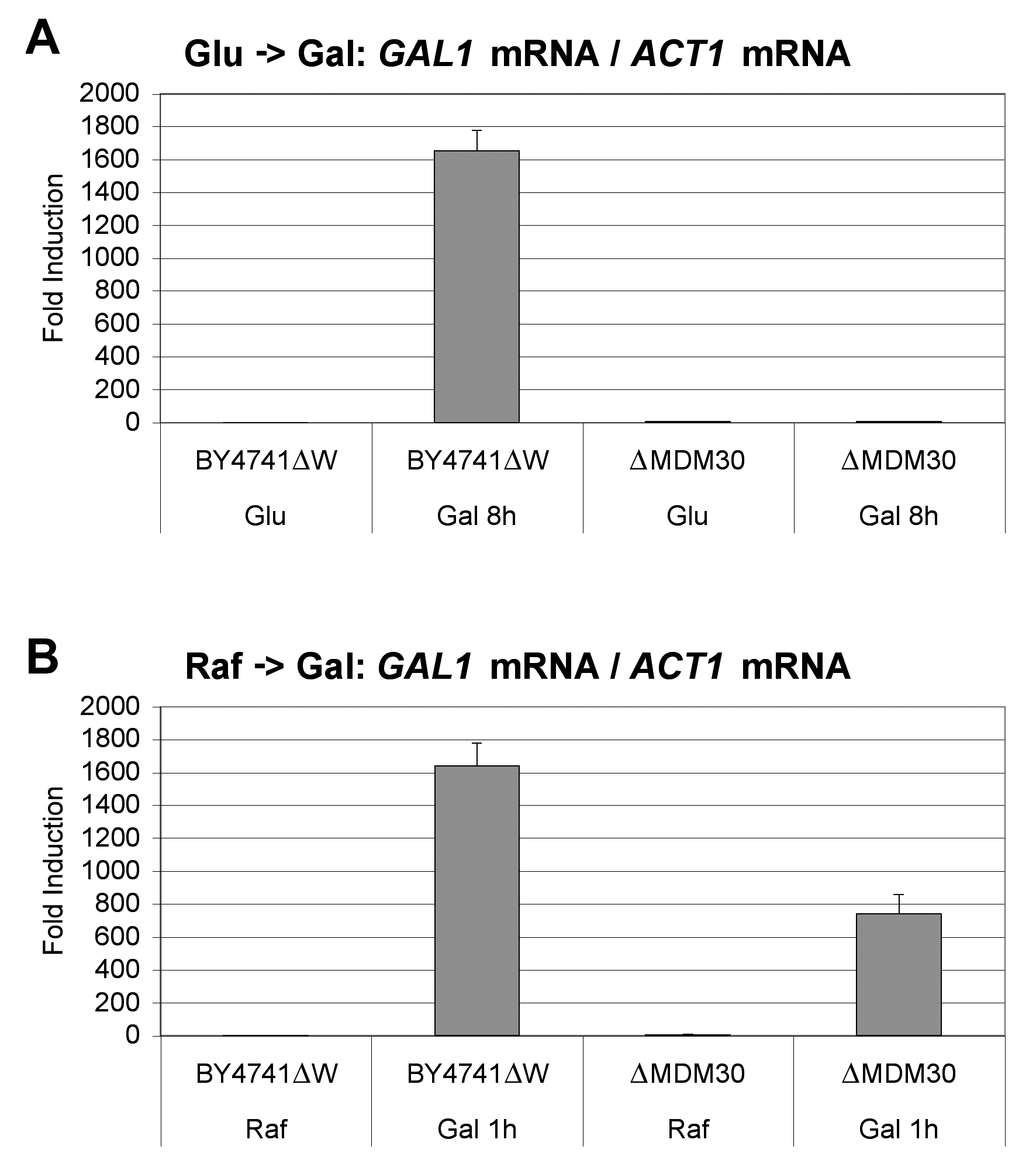

Supplement: Figure S13 — Galactose induction of GAL1 mRNA is restored in the ΔMDM30 strain, if the cells are pre-grown with raffinose instead of with glucose. (A) BY4741ΔW wild-type and ΔMDM30 cells were grown in glucose liquid media to OD600 nm = 1 (Glu) and induced with galactose liquid media for 8 h (Gal 8 h). Total RNA was isolated and GAL1 mRNA was determined relative to ACT1 mRNA by quantitative real-time PCR. The value determined for BY4741ΔW wild-type cells grown with glucose liquid media was set as 1 and the error bars indicate the standard deviations between three replicates. (B) BY4741ΔW wild-type and ΔMDM30 cells were grown in raffinose liquid media to OD600 nm = 1 (Raf) and induced with galactose liquid media for 1 h (Gal 1 h). Total RNA was isolated and GAL1 mRNA was determined relative to ACT1 mRNA by quantitative real-time PCR. The value determined for BY4741ΔW wild-type cells grown with raffinose liquid media was set as 1 and the error bars indicate the standard deviations between three replicates. (TIF) [file pbio.1001290.s013.tif]

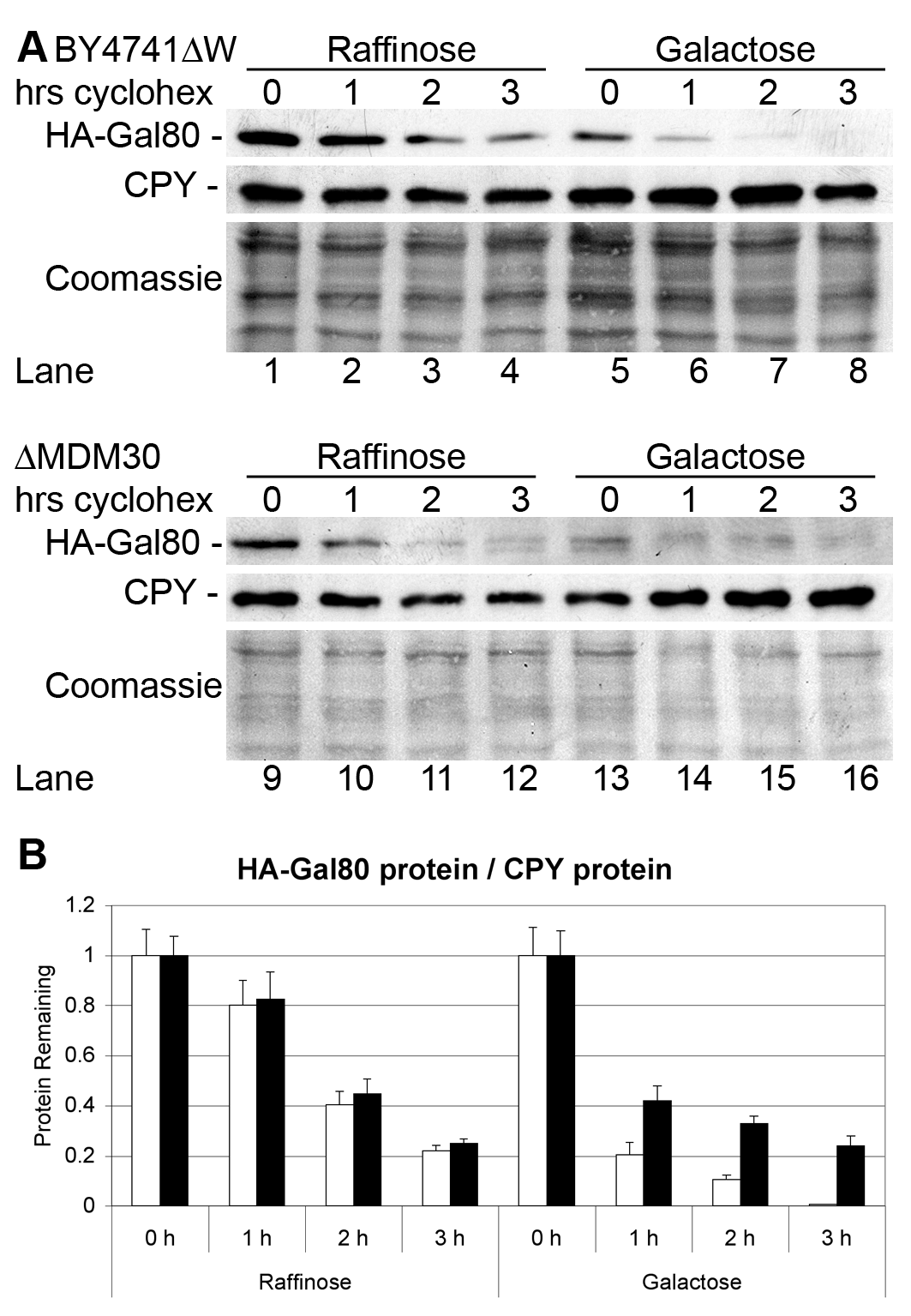

Supplement: Figure S14 — Protein degradation of Gal80 is restored in the ΔMDM30 strain, if the cells are pre-grown with raffinose instead of with glucose. (A) HA-tagged Gal80 was expressed in BY4741ΔW and BY4741ΔWΔMDM30 cells from the single-copy vector RS316 under the control of the ACT1 promoter. Cells were grown in raffinose liquid media (lanes 1 to 4 and 9 to 12) to OD600 nm = 1 and induced with galactose liquid media for 1 h (lanes 5 to 8 and 13 to 16). Cycloheximide was added at time = 0 and the amount of Gal80 protein remaining in the cells after the indicated number of hours was determined by Western blot with the help of an anti-HA antibody (upper panels). The membranes were stripped and reprobed with an anti-CPY antibody (middle panels), followed by a second stripping and staining with Coomassie Blue as loading controls (lower panels). (B) The ratio of the amount of HA-Gal80 protein to CPY protein in BY4741ΔW cells (white bars) and BY4741ΔWΔMDM30 cells (black bars) for each time point in part A was determined with Image J. The ratio of the band intensities before the addition of cycloheximide (time = 0) was set as 1 and the error bars indicate the deviations between duplicates. (TIF) [file pbio.1001290.s014.tif]

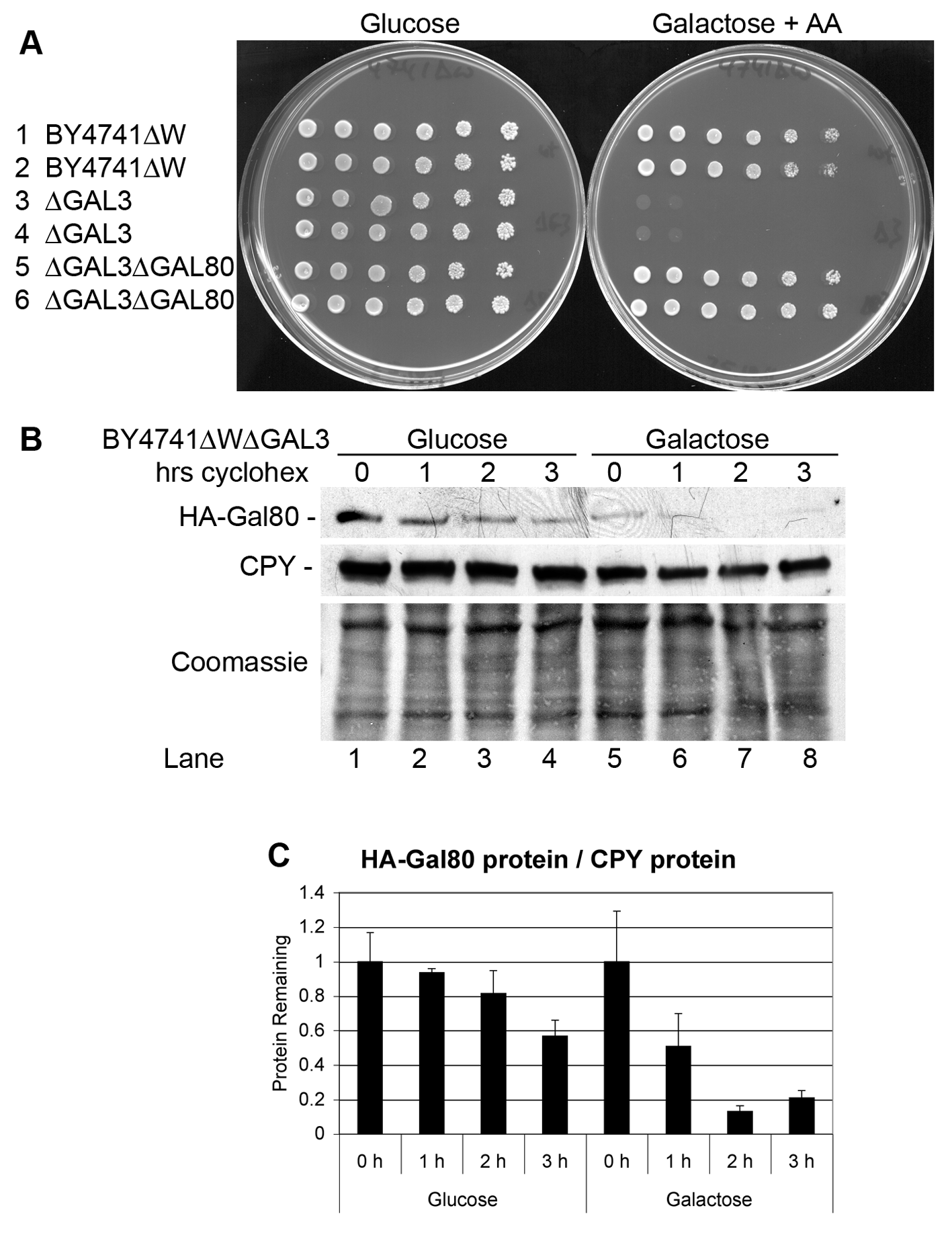

Supplement: Figure S15 — Gal3 is not required for the galactose-stimulated protein degradation of Gal80. (A) BY4741ΔW cells of the indicated genotype were 10-fold serially diluted, dropped onto the depicted plates, and incubated at 28°C for 3 d. The titrations were performed in duplicates. The Galactose+AA plate contained 1 mg/l Antimycin A. (B) HA-tagged Gal80 was expressed in BY4741ΔWΔGAL3 cells from the single-copy vector RS316 under the control of the ACT1 promoter. Cells were grown in glucose liquid media (lanes 1 to 4) to OD600 nm = 1 and induced with galactose liquid media for 1 h (lanes 5 to 8). Cycloheximide was added at time = 0 and the amount of Gal80 protein remaining in the cells after the indicated number of hours was determined by Western blot with the help of an anti-HA antibody (upper panel). The membranes were stripped and reprobed with an anti-CPY antibody (middle panel), followed by a second stripping and staining with Coomassie Blue as loading control (lower panel). (C) The ratio of the amount of HA-Gal80 protein to CPY protein for each time point in part B was determined with Image J. The ratio of the band intensities before the addition of cycloheximide (time = 0) was set as 1 and the error bars indicate the deviations between duplicates. (TIF) [file pbio.1001290.s015.tif]

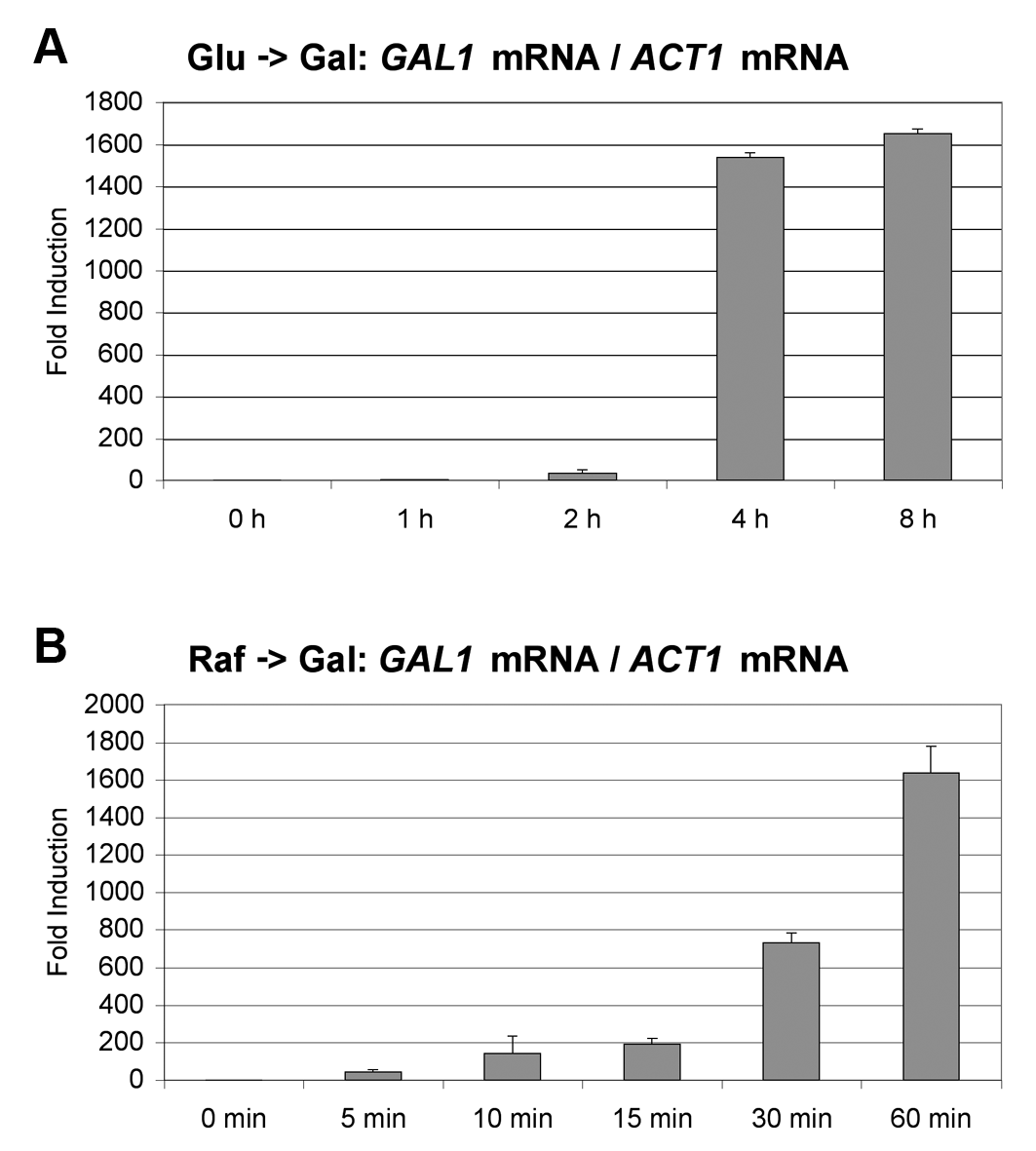

Supplement: Figure S16 — The degradation of Gal80 as the limiting factor for the activation of the GAL1 gene. (A) BY4741ΔW cells were grown in glucose liquid media to OD600 nm = 1 (0 h) and induced with galactose liquid media for the indicated number of hours. Total RNA was isolated and GAL1 mRNA was determined relative to ACT1 mRNA by quantitative real-time PCR. The value determined for BY4741ΔW cells grown with glucose liquid media was set as 1 and the error bars indicate the standard deviations between three replicates. (B) BY4741ΔW cells were grown in raffinose liquid media to OD600 nm = 1 (0 min) and induced with galactose liquid media for the indicated number of minutes. Total RNA was isolated and GAL1 mRNA was determined relative to ACT1 mRNA by quantitative real-time PCR. The value determined for BY4741ΔW cells grown with raffinose liquid media was set as 1 and the error bars indicate the standard deviations between three replicates. (TIF) [file pbio.1001290.s016.tif]
