# Supplementary material for: Mediator Acts Upstream of the Transcriptional Activator Gal4
Source: PLoS Biol. 2012 Mar 27;10(3):e1001290. doi: 10.1371/journal.pbio.1001290 (PMC3313914; doi:10.1371/journal.pbio.1001290)
Supplement: Table S1 — Genotypes and sources of strains used in this study. (DOC) [file pbio.1001290.s017.doc]

Table S1. Genotypes of Strains.

| Strain | Genotype | Source |
| --- | --- | --- |
| SUB288+pUB100 | *MATa; lys2-801; leu2-3,112; ura3-52; his3Δ200; trp1-1; ubi1::TRP1; ubi2-Δ2:: ura3; ubi3-Δub2; ubi4-Δ2::LEU2+pUB100* | [53] |
| *SUB288ΔWL+316-Ub* | *SUB288;TRP1::hisG; LEU2::hisG+316-Ub* | [41] |
| *SUB288GAL3ΔWL+316-Ub* | *SUB288; TRP1::hisG; LEU2::hisG; gal3::GAL3(PCR)+316-Ub* | This study |
| *SUB288GAL3ΔL+316-Ub* | *SUB288; TRP1::hisG; LEU2::hisG; gal3::YIplac204-GAL3+316-Ub* | This study |
| *SUB288GAL3ΔGAL80ΔWL*  *+316-Ub* | *SUB288; TRP1::hisG; LEU2::hisG; gal3::GAL3(PCR);GAL80::hisG+316-Ub* | This study |
| *SUB288ΔMDM30ΔW+316-Ub* | *SUB288ΔWL; MDM30::LEU2+316-Ub* | This study |
| *JD52* | *MATa; ura3-52; leu2-3,112; his3Δ200; lys2-801; trp1Δ63* | [58] |
| *JD52SKP1wt* | *JD52; SKP1::HIS3+Nub-Skp1wt* | [37] |
| *JD52skp1dM* | *JD52;SKP1::HIS3+Nub-Skp1V90A,E129A* | [37] |
| *JD52skp1dMΔGAL80* | *JD52skp1dM; GAL80::hisG* | This study |
| *JD52skp1dMΔMIG2* | *JD52skp1dM; MIG2::hisG* | [37] |
| *BY4741* | *MATa; his3Δ1; leu2Δ0; met15Δ0; ura3Δ0* | EUROSCARF |
| *BY4741ΔW* | *BY4741; TRP1::hisG* | [43] |
| *BY4741ΔDAS1* | *BY4741; YJL149W::kanMX4* | EUROSCARF |
| *BY4741ΔDAS1ΔW* | *BY4741ΔDAS1; TRP1::hisG* | [37] |
| *BY4741ΔDAS1ΔGAL80ΔW* | *BY4741ΔDAS1ΔW; GAL80::hisG* | This study |
| *BY4741ΔUFO1* | *BY4741; YML088W::kanMX4* | EUROSCARF |
| *BY4741ΔUFO1ΔW* | *BY4741ΔUFO1; TRP1::hisG* | [37] |
| *BY4741ΔUFO1ΔGAL80ΔW* | *BY4741ΔUFO1ΔW; GAL80::hisG* | This study |
| *BY4741ΔMDM30* | *BY4741; YLR368W::kanMX4* | EUROSCARF |
| *BY4741ΔMDM30ΔW* | *BY4741ΔMDM30; TRP1::hisG* | This study |
| *BY4741ΔMDM30ΔGAL80ΔW* | *BY4741ΔMDM30ΔW; GAL80::hisG* | This study |
| *BY4741ΔGAL3* | *BY4741; YDR009w::kanMX4* | EUROSCARF |
| *BY4741ΔGAL3ΔW* | *BY4741ΔGAL3; TRP1::hisG* | This study |
| *BY4741ΔGAL11* | *BY4741; YOL051W::kanMX4* | EUROSCARF |
| *BY4741ΔGAL11ΔW* | *BY4741ΔGAL11; TRP1::hisG* | [43] |
| *BY4741ΔGAL11ΔGAL80ΔW* | *BY4741ΔGAL11ΔW; GAL80::hisG* | This study |
| *BY4741ΔSNF4* | *BY4741; YGL115W::kanMX4* | EUROSCARF |
| *BY4741ΔSNF4ΔW* | *BY4741ΔSNF4; TRP1::hisG* | This study |
| *BY4741ΔSNF4ΔGAL80ΔW* | *BY4741ΔSNF4ΔW; GAL80::hisG* | This study |
| *BY4741ΔW::128-Skp1-HA_3_H_10_* | *BY4741ΔW::YIplac128-Skp1c-HA_3_H_10_* | This study |
| *BY4742* | *MATα; his3Δ1; leu2Δ0; lys2Δ0; ura3Δ0* | EUROSCARF |
| *BY4742ΔW* | *BY4742; TRP1::hisG* | [43] |
| *BY4742ΔSNF1* | *BY4742; YOL051W::kanMX4* | EUROSCARF |
| *BY4742ΔSNF1ΔW* | *BY4742ΔSNF1; TRP1::hisG* | [37] |
| *BY4742ΔSNF1ΔGAL80ΔW* | *BY4742ΔSNF1ΔW; GAL80::hisG* | This study |
| *BY4742ΔW::128-Snf1-HA_3_H_10_* | *BY4742ΔW::YIplac128-Snf1c-HA_3_H_10_* | This study |
| *BY4742ΔSRB10* | *BY4742; YPL042c::kanMX4* | EUROSCARF |
| *BY4742ΔSRB10ΔGAL80* | *BY4742ΔSRB10; GAL80::hisG* | This study |
| *BY4742ΔSRB11* | *BY4742; YNL025c::kanMX4* | EUROSCARF |
| *BY4742ΔSRB11ΔGAL80* | *BY4742ΔSRB11; GAL80::hisG* | This study |
