# Supplementary material for: Mediator Acts Upstream of the Transcriptional Activator Gal4
Source: PLoS Biol. 2012 Mar 27;10(3):e1001290. doi: 10.1371/journal.pbio.1001290 (PMC3313914; doi:10.1371/journal.pbio.1001290)
Supplement: Table S2 — Names of plasmids constructed for this study and DNA sequences of PCR primers used to generate these plasmids. (DOC) [file pbio.1001290.s018.doc]

Table S2. Sequences of primers.

| Construct | PCR Primers |
| --- | --- |
| *pSuper-siSRB7* | gatcccctgaatcagctttaaagcatttcaagagaatgctttaaagctgattcatttttg tcgacaaaaatgaatcagctttaaagcattctcttgaaatgctttaaagctgattcaggg |
| *pSuper-siSKP1* | gatccccgcactgctctgtttataatttcaagagaattataaacagagcagtgctttttg tcgacaaaaagcactgctctgtttataattctcttgaaattataaacagagcagtgcggg |
| *hSrb7-Cub-RUra314* | GCCCAATTGAAAATGGCGGATCGGCTCACGCA  GCCGCCTCGAGGCGTTTGAGTCTGGAAGGAGACTGGC |
| *GEX-5X-1-GST-hSrb7*  *PET11a-H6-HA-hSrb7* | GCCCAATTGAAAATGGCGGATCGGCTCACGCA  GCCGCCTCGAGCTATGAGTCTGGAAGAGACT |
| *GEX-5X-1-GST-hSkp1*  *CMV-myc-hSkp1* | GCCGGATCCCAATTGATGCCTTCAATTAAGTTGCA  GCCGCCGTCGACTCACTTCTCTTCACACCACT |
| *Srb7-Cub-RUra314* | GCCGAATTCCATATGACAGATAGATTAACACAA  GCCGCCGTCGACGTGCTCTTTTTTGAGTTTGCA |
| 317-Ub | GCCGAATTCATGCAGATTTTCGTCAAGACT  GCCGCGGCCGCTAACCACCTCTTAGCCTTA |
| *317-H10-Ub* | GCCGGGCCCTCTACCTTGCAGACCCATATA  gccgaattcgtggtgatgatggtgatgatggtgatgatgcattttagtaaattttcgatcttggg |
| *317-H10-UbD58A* | TGAATGTTGTAAGCAGACAGCGTTC  GAACGCTGTCTGCTTACAACATTCA |
| *NKY51-GAL80, 316‑HA-Gal80, 424-GST‑Gal80* | GCCCAATTGCCCATGGACTACAACAAGAGAT GCCGCGTCGACAAACTATAATGCGAGATAT |
| *Pact314-Alpha2*  *Pact315-Alpha2* | GCCGAATTCGAAATGAATAAAATACCCATTAAA GCCGTCGACTCATTCTTTCTTCTTTGCCAG |
| Pact314-Gal3 *Pact315-Gal3* | GCCGAATTCAAAATGAATACAAACGTTCCA GCCGCCGTCGACTTATTGTTCGTACAAACA |
| SRB7::HIS3  (PCR Fragment) | aagagggacataacatttcactagttccaatacattatatgctcttttaacaatgacagaagcagaaagcc atagtacattttctgtcctctgttcgcaaatttctttgtaaaacttaagtctacataagaacaccttt |
| *YCplac22-SRB7*  *YCplac33-SRB7* | CGCCTCTAGAAGCTTCGATGATGTTCTTTATTCT  CGCGGAATTCGATCATTTTCATGTGCGTGC |
| YG1u-GST-Srb7Δ40 | GCCGAATTCATGTCCGATAAGCATGCC  GCCGCCATGCATTATGTGCTCTTTTTTGAGTT |
| *316-HA-Gal80ΔN6* | GCCCAATTGTCTTCGGTCTCAACCGTG  GCCGCGTCGACAAACTATAATGCGAGATAT |
| *316-HA-Gal80ΔN8* | GCCCAATTGGTCTCAACCGTGCCTAAT  GCCGCGTCGACAAACTATAATGCGAGATAT |
| *316-HA-Gal80ΔN10* | GCCCAATTGACCGTGCCTAATGCAGCT  GCCGCGTCGACAAACTATAATGCGAGATAT |
| *316-HA-Gal80ΔN12* | GCCCAATTGCCTAATGCAGCTCCCATA  GCCGCGTCGACAAACTATAATGCGAGATAT |
| *316-HA-Gal80ΔN14* | GCCCAATTGGCAGCTCCCATAAGAGTC  GCCGCGTCGACAAACTATAATGCGAGATAT |
| *316-HA-Gal80ΔN16* | GCCCAATTGCCCATAAGAGTCGGATTC  GCCGCGTCGACAAACTATAATGCGAGATAT |
| *316-HA-Gal80ΔN18* | GCCCAATTGAGAGTCGGATTCGTCGGT  GCCGCGTCGACAAACTATAATGCGAGATAT |
| *316-HA-Gal80ΔN20* | GCCCAATTGGGATTCGTCGGTCTCAAC  GCCGCGTCGACAAACTATAATGCGAGATAT |
| *423-HA3-Mdm30* | GCCCAATTGATGACAAAGAGGAGAAAC GCCGTCGACTATAAATTATGTAAA |
| *423-HA3-Das1* | GCCGAATTCATGCCATTTCAAGATTAT  GCCGCGGCCGCTTACATAACAGTGGCTAT |
| *423-HA3-Ufo1* | GCCGAATTCATGGAGCGGCCTGGCTTG  GCCGCGGCCGCTCAATTGATTTCACTCAA |
| *YIplac128-Snf1c-HA_3_-H_10_* | GCCGAATTCATGAGCAGTAACAACAAC  GCCGTCGACGCATTGCTTTGACTGTTAAC |
| *YIplac128-Skp1c-HA_3_-H_10_* | GCCCAATTGATGGTGACTTCTAATGTTGT  GCCGTCGACGCGTGACGGTCTTCAGCCCA |
